# PASylation: a biological alternative to PEGylation for extending the plasma half-life of pharmaceutically active proteins

**DOI:** 10.1093/protein/gzt023

**Published:** 2013-06-10

**Authors:** Martin Schlapschy, Uli Binder, Claudia Börger, Ina Theobald, Klaus Wachinger, Sigrid Kisling, Dirk Haller, Arne Skerra

**Affiliations:** 1 Munich Center for Integrated Protein Science (CIPS-M) & Lehrstuhl für Biologische Chemie, Technische Universität München, Emil-Erlenmeyer-Forum 5, 85350 Freising-Weihenstephan, Germany; 2 XL-protein GmbH, Lise-Meitner-Str. 30, 85354 Freising, Germany; 3 Chair of Nutrition and Immunology & ZIEL–Research Center for Nutrition and Food Sciences, Biofunctionality Unit, Technische Universität München, 85350 Freising-Weihenstephan, Germany

**Keywords:** biologic, dosing, kidney filtration, pharmacokinetics, therapeutic protein

## Abstract

A major limitation of biopharmaceutical proteins is their fast clearance from circulation via kidney filtration, which strongly hampers efficacy both in animal studies and in human therapy. We have developed conformationally disordered polypeptide chains with expanded hydrodynamic volume comprising the small residues Pro, Ala and Ser (PAS). PAS sequences are hydrophilic, uncharged biological polymers with biophysical properties very similar to poly-ethylene glycol (PEG), whose chemical conjugation to drugs is an established method for plasma half-life extension. In contrast, PAS polypeptides offer fusion to a therapeutic protein on the genetic level, permitting *Escherichia coli* production of fully active proteins and obviating *in vitro* coupling or modification steps. Furthermore, they are biodegradable, thus avoiding organ accumulation, while showing stability in serum and lacking toxicity or immunogenicity in mice. We demonstrate that PASylation bestows typical biologics, such as interferon, growth hormone or Fab fragments, with considerably prolonged circulation and boosts bioactivity *in vivo*.

## Introduction

Short plasma half-life in humans of just a few hours after intravenous (i.v.) bolus injection is a typical feature of almost all biopharmaceuticals (Kontermann, 2012), or corresponding drug candidates, with renal clearance providing the predominant route of fast elimination. According to the rules of allometric scaling (Mahmood, 2005) this translates into loss from circulation already few minutes after application to mice—the preferred laboratory animal—which is often insufficient to achieve a solid pharmacodynamic (PD) effect and constitutes a major handicap for preclinical drug development. A remarkable exception are intact IgG antibodies, which show a half-life of 1–3 weeks in humans (Lobo *et al.*, 2004) and 6–8 days in mice (Vieira and Rajewsky, 1988; Milenic *et al.*, 2010), owing to their large size as well as FcRn-mediated endosomal recycling (Roopenian and Akilesh, 2007).

PEGylation, i.e. chemical coupling with the synthetic polymer poly-ethylene glycol (PEG), has emerged as an accepted technology for the development of biologics that exercise prolonged action, with around 10 clinically approved protein and peptide drugs to date (Jevsevar *et al.*, 2010). PEG forms a highly solvated, structurally disordered random chain in aqueous solution which, if conjugated, leads to an expanded hydrodynamic volume for the pharmaceutically active biomolecule and, consequently, to strongly retarded glomerular filtration. This phenomenon depends on the length of the PEG chain and is essentially based on a pure biophysical size effect (Pasut and Veronese, 2007).

While conceptionally simple and effective at first glance, PEGylation shows a growing number of drawbacks (Gaberc-Porekar *et al.*, 2008; Knop *et al.*, 2010) that not only hamper clinical drug as well as bioprocess development but also limit its applicability for drug discovery and biomedical research: (i) the necessity of chemical *in vitro* coupling and corresponding modification that has to be made to the protein of interest; (ii) the frequent loss in bioactivity of the biological; (iii) the high cost and inherent polydispersity of commercially available activated PEG derivatives and associated difficulties in product analysis; (iv) the poor bioavailability of subcutaneously (s.c.) administered PEGylated proteins due to the waxy behavior of highly concentrated solutions; (v) accumulating evidence on the immunogenicity of PEG (Garay *et al.*, 2012); and (vi) the lacking biodegradability of the unnatural PEG polymer, which can lead to tissue accumulation such as renal tubular vacuolation (Bendele *et al.*, 1998).

In particular, the chemical coupling procedure for PEG is a delicate step, which requires profound knowledge of protein chemistry and often involves laborious optimization. While coupling to Lys side chains via *N*-hydroxysuccinimide (NHS)-activated PEG compounds is technically straightforward, it results in poorly defined product mixtures (Foser *et al.*, 2003), both in terms of coupling stoichiometry and of site-specificity, and often leads to impaired activity (Bailon *et al.*, 2001) (i.e. reduced affinity for the receptor or target). Site-specific coupling, on the other hand, requires the introduction of a unique reactive side chain into the protein of interest, usually a Cys residue, which involves extensive positional optimization (Rosendahl *et al.*, 2005) in order to preserve functional activity and prevent interference with intrinsic disulfide bonds, which are present in most therapeutic proteins of interest. In fact, this kind of initial protein modification together with the subsequent PEG coupling step usually lowers the product yield by a considerable extent.

In search for a biological alternative to PEGylation, we designed genetically encodable amino acid sequences that form a natively unstructured polypeptide chain with high solubility and, importantly, lack of charges, thus offering comparable biophysical properties as PEG. Initially, we investigated Gly-based polypeptides (Schlapschy *et al.*, 2007) which, as expected, exhibited random conformation as part of fusion proteins—e.g. with a recombinant Fab fragment—and led to an enlarged hydrodynamic volume. However, despite detectably prolonged circulation in mice, the half-life extending effect of these Gly-rich sequences with lengths up to 200 residues appeared too low for most biomedical applications whereas their inherently limited solubility prevented the preparation of longer chains.

**Fig. 1.**
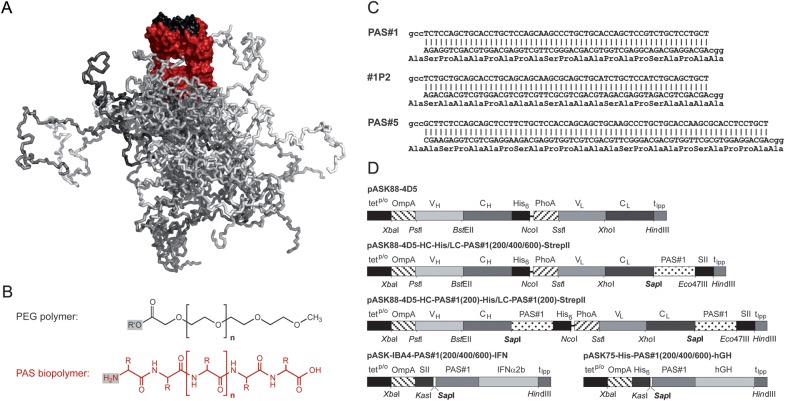
Concept of PASylation. (**A**) Modelled structure of the PASylated Fab fragment of an antibody. Both Ig chains are colored red whereas the antigen-binding site is shown in black. Twenty-four arbitrarily selected random conformations of the PAS polypeptide tag at the *C*-terminus of the light chain are presented as snapshots and shown in different shades of grey in order to illustrate the fluctuating random coil-like, space-filling behavior. (**B**) Comparison of the chemical composition between the synthetic linear polymer PEG (potentially carrying a reactive group R′ at the left end) with its hydrophilic ether oxygen bridges and an unfolded biological polypeptide with its polar peptide groups (side chains abbreviated with R). (**C**) Nucleotide and encoded amino acid sequences of the building blocks for PAS#1, PAS#1P2 and PAS#5 gene cassettes obtained by hybridization of two complementary oligodeoxynucleotides, giving rise to two non-palindromic sticky ends that can be mutually ligated or cloned via a *Sap*I restriction site. (**D**) Schematic representation of expression cassettes on the plasmids used in this study with coding elements and singular restriction sites indicated. All genes are under control of the chemically inducible *tet*^p/o^ while the vector backbone corresponds to pASK75 (Skerra, 1994).

Since homo-polymers of other small amino acids are known to adopt secondary structure instead of a random coil (Schlapschy *et al.*, 2007), we subsequently explored combinations of different residues. Surprisingly, we found that sequences comprising the three small amino acids Pro, Ala and Ser (PAS) allow the efficient biosynthesis of long hydrophilic and highly soluble polypeptide chains exhibiting properties remarkably similar to PEG (Fig. 1). Here, we describe the preparation of genetic conjugates with several types of therapeutic proteins as well as their biochemical and biophysical characterization, and we demonstrate the strong effect of PASylation with regard to prolonged pharmacokinetics (PK) and enhanced *in vivo* activity.

## Results

### Design of PAS sequences and corresponding gene cassettes

Our concept for the design of polypeptides with PEG-like properties such as, in particular, large hydrodynamic volume, high solubility, and lack of charges was based on a hypothetical l-α-amino acid sequence whose backbone should adopt robust random chain conformation in aqueous solution and at ambient or body temperature and whose side chains have low tendency to form intermolecular interactions other than with solvent molecules. To achieve this objective, all natural amino acids with hydrophobic or charged side chains had to be neglected. Of the remaining hydrophilic amino acids, those with carboxamide side chains, i.e. Asn and Gln, were excluded because of their known tendency to form aggregates and their role in protein folding pathologies, for example, Huntington's disease (Furukawa *et al.*, 2009). Furthermore, the β-branched amino acid Thr was omitted due to its pronounced β-sheet propensity while His with its imidazole side chain was rejected because of its basic behavior and metal-ion binding capacity. Finally, the smallest amino acid, Gly, was avoided on the grounds of its poor solubility with increasing chain length and its generally compacting effect on the random coil structure of unfolded polypeptides (Cantor and Schimmel, 1980).

Consequently, only the three small amino acids Ala, Ser and Pro, which all are fairly hydrophilic—with octanol/water partition coefficients close to the side chain lacking reference amino acid Gly (Creighton, 1993)—remained as a preferred set of building blocks. Notably, however, homo-polymers of each of these amino acids are known to form stable secondary structures: α-helix in the case of Ala (Shental-Bechor *et al.*, 2005), β-sheet in the case of Ser (Quadrifoglio and Urry, 1968) and a type II *trans* helix (Stapley and Creamer, 1999) in the case of poly-Pro. Nevertheless, we speculated that appropriate mixtures thereof might form the aspired random coil polypeptides for two reasons: first, the individually differing secondary structure propensities of Pro, Ala and Ser should cancel out each other and, second, the imino acid Pro is well known for its *N*-terminal *cis*/*trans* isomerization (Yaron and Naider, 1993) which provides an additional degree of freedom and raises the conformational entropy of the unfolded polypeptide backbone. Finally, we took care that the genetically defined PAS sequences exhibited an erratic order (Fig. 1C), thus avoiding two-, three- or four-residue repeats as they typically occur in secondary structures such as β-sheet, α-helix or the collagen triple helix (Creighton, 1993).

Employing some of the resulting sequence motifs, we designed suitable gene cassettes using highly translated *Escherichia coli* codons. To this end, pairs of complementary oligodeoxynucleotides (typically encoding a 20- or 24-residue stretch) that upon hybridization lead to two 5′-protruding, non-palindromic three-nucleotide overhangs—corresponding to an Ala codon (GCC)—were subjected to unidirectional ligation (Fig. 1C). In this way longer synthetic gene fragments (Schlapschy *et al.*, 2007), typically encoding 200 or 192 residues, were obtained and subcloned on a suitable vector for sequence verification and further use. With this repetitive design uniform biophysical properties along the entire PAS polypeptide were maintained, in spite of the non-periodic sequence within each short minimal cassette.

### Functional production of biomedically relevant PAS fusion proteins in *E.coli*

To investigate the biophysical properties of PAS amino acid sequences and their effects on plasma half-life *in vivo*, three model proteins representing different fold types were chosen: (i) a recombinant Fab fragment of the humanized anti-HER2 antibody 4D5 (trastuzumab, Carter *et al.*, 1992; Fab), (ii) human interferon α2b (Borden *et al.*, 2007; IFN), and (iii) human growth hormone (somatropin, Franklin and Geffner, 2009; hGH).

In case of the 4D5 Fab fragment the PAS polypeptides #1 and #5 were fused to the *C*-terminus of the immunoglobulin (Ig) light chain, similarly as previously described for the Gly-rich sequences (Schlapschy *et al.*, 2007). Using a plasmid for the periplasmic secretion of the Fab fragment, on which the heavy chain fragment was equipped with a His_6_-tag, the *Strep*-tag II was appended to the *C*-terminus of the light chain and a single type IIS *Sap*I restriction site (generating a sticky end corresponding to an Ala codon, see above) was introduced in between, right downstream of the *C*-terminal Cys residue of the C_κ_ domain (Fig. 1D). The gene cassettes for the PAS polypeptides #1 and #5 were then inserted. As a consequence of this cloning strategy, the restriction site at the downstream end of the inserted PAS(200) cassette was lost whereas the one at the upstream end was retained, subsequently allowing insertion of additional cassettes in a consecutive manner, thus yielding PAS(400), PAS(600) or even longer fusion proteins.

In another strategy, a second *Sap*I restriction site was introduced directly downstream of the *C*-terminal Cys residue of the C_H_1 domain. Thus, after insertion of a second PAS(200) gene cassette, a Fab fragment with two separate PAS tags was prepared (dubbed duoPAS Fab), each at the *C*-termini of its light and heavy chains (Fig. 1D), in effect resembling a branched PEG chain (Jevsevar *et al.*, 2010). Finally, for comparison, a conventionally PEGylated Fab fragment was prepared via site-specific maleimide coupling of branched PEG(20)_2_ to a free Cys residue at the *C*-terminus of the light chain.

**Fig. 2.**
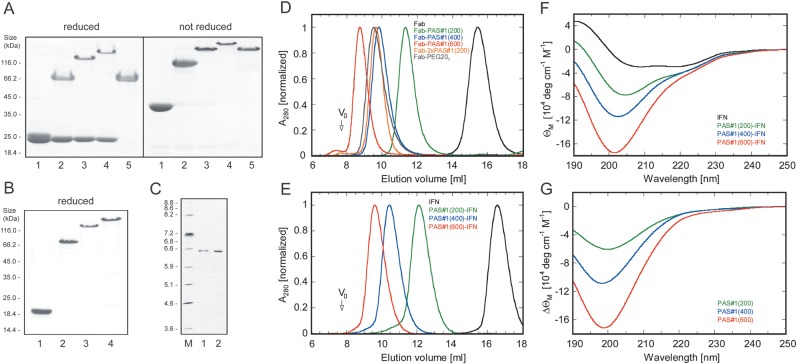
Biochemical and biophysical analysis of PAS fusion proteins. (**A**) Analysis of the recombinant 4D5 Fab fragment and its PASylated versions, all purified from the periplasmic cell fraction of *E.coli*, by Coomassie-stained 12% SDS–PAGE. Lane 1, the original Fab fragment; lanes 2–4, its fusions with PAS#1(200), PAS#1(400) and PAS#1(600), respectively; lane 5, the Fab fragment carrying a PAS#1(200) polymer at both of its light and heavy chains. Samples on the right are the same but were not reduced with 2-mercaptoethanol. (**B**) Analysis of recombinant IFN and its PASylated versions as in (A). Lane 1, original IFN; lanes 2–4, its fusions with PAS#1(200), PAS#1(400) and PAS#1(600), respectively. (**C**) Isoelectric focussing (IEF) of IFN and PAS#1(600)-IFN. Lane M, IEF marker proteins; lane 1, recombinant IFN; lane 2, PAS#1(600)-IFN. Both proteins show single bands at almost the same position corresponding to pH ∼6.6, in line with the calculated p*I* of 6.51 for IFN. (**D**) Analysis of hydrodynamic volume for the purified recombinant Fab fragment and its PASylated versions by SEC (peaks normalized). Two hundred and fifty microliters (250 µl) of each protein at a concentration of 0.25 mg/ml was applied to a Superdex S200 10/300 GL column equilibrated with PBS. The arrow indicates the void volume of the column (7.8 ml). (**E**) Analytical SEC of IFN and its PASylated versions as in (D). (**F**) CD spectra of IFN and its PASylated versions. Spectra were recorded at room temperature in 50 mM K_2_SO_4_, 20 mM K-P_i_ pH 7.5 using a 0.01 cm quartz cuvette and normalized to the molar ellipticity (Θ_M_) for each protein. (**G**) Molar difference CD spectra for the PASylated IFN versions with 200, 400 and 600 residues obtained by subtracting the spectrum of the original IFN from (F).

All Fab-PAS fusion proteins were produced in *E.coli* via periplasmic secretion in a soluble state and purified by means of His_6_-tag and *Strep*-tag II as well as size exclusion chromatography (SEC), resulting in homogeneous functional protein preparations (Fig. 2). In all Fab constructs the interchain disulfide bridge was properly formed, with ≥95% covalent linkage between light and heavy chains, hence indicating no steric hindrance in Ig chain association due to the presence of the voluminous PAS tag, even for the duoPAS Fab format. Interestingly, those chains fused with the PAS tag revealed a drastically reduced electrophoretic mobility in sodium dodecyl sulfate polyacrylamide gel electrophoresis (SDS–PAGE) depending on the polymer length: e.g. the light chain fused with PAS(200) migrated at ∼70 kDa compared with a calculated mass of 41.4 kDa, while a PAS(400) tag led to an apparent size of 150 kDa (calc. 57.9 kDa). This behavior indicates reduced binding of SDS which normally provides the electrophoretic driving force with its negatively charged sulfate head group. Furthermore, with increasing PAS polypeptide length the protein band showed less efficient Coomassie staining, in line with the absence of hydrophobic and/or basic residues that otherwise are responsible for binding of this dye (Georgiou *et al.*, 2008). Electrospray ionization mass spectrometry (ESI-MS) confirmed in each case the expected molecular mass and, notably, monodisperse composition, without indication of prematurely terminated gene products (Supplementary Fig. S1).

In case of IFN and hGH the PAS polypeptide was fused to the *N*-termini of the therapeutic proteins. Using a single *Sap*I restriction site between the *N*-terminal *Strep*-tag II and IFN or between the His_6_-tag and hGH, individual 200-residue PAS gene cassettes were inserted in a consecutive manner as described above (Fig. 1D). Both proteins fused with PAS tags of up to 600 residues were again produced in *E.coli* via periplasmic secretion, to ensure proper disulfide bond formation, and purified to homogeneity as above. During SDS–PAGE analysis a similar retarding effect of PASylation on the electrophoretic mobility was observed as for the Fab (Fig. 2B and Supplementary Fig. S2).

### Biophysical properties: hydrodynamic volume and secondary structure analysis of PAS fusion proteins

The biophysical properties of the PAS polypeptides were first characterized with the 4D5 Fab and IFN (having native molecular masses of 48.0 and 21.0 kDa, respectively). The effect of the PAS#1 polypeptide on the hydrodynamic volume of corresponding fusion proteins was quantitatively analyzed by SEC. For each of the purified PASylated Fab fragments a single homogenous elution peak was observed (Fig. 2D). Notably, the elution volume strongly decreased with increasing length of the polymer (Table I), leading to an apparent molecular size of up to 691 kDa for the PAS#1(600) Fab (calc. 99.0 kDa). Thus, although the real increase in mass upon PASylation of the recombinant Fab fragment was just 51 kDa the apparent mass increase was 660 kDa, i.e. 22-fold. This observation clearly indicates the massive effect of a larger hydrodynamic volume that has to be expected if the appended PAS polypeptide assumes random coil structure (Squire, 1981). In comparison, the Fab-PEG(20)_2_ (with a calculated mass of 88.1 kDa) showed an apparent mass of 478 kDa, which was significantly lower and actually closer to the one of the linear PAS#1(400) Fab with an apparent mass of 451 kDa (calc. 82.5 kDa). Comparable results were obtained for PASylated IFN (Fig. 2E). In this case the apparent molecular size of the PAS#1(600) fusion protein was 610 kDa, corresponding to an increase in hydrodynamic volume by a factor 26 due to the attached random coil polypeptide.

**Table I.** Biophysical and *in vivo* murine PK data of PASylated proteins

Protein sample	Mass (kDa)	SEC size (kDa)	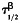 (h)	AUC (h μg ml^−1^)	CL (ml h^−1^ kg^−1^)	PK factor	k_on_ (M^−1^ s^−1^)	k_off_ (s^−1^)	*K*_D_ (nM)
Fab	48.0	31	1.34	17.03	293.56	1	7.63 × 10^5^	8.72 × 10^−5^	0.114 ± 0.009
Fab-PAS#1(100)	56.5	117.3	2.71	123.39	40.52	2.0	–	–	–
Fab-PAS#1(200)	66.0	219	5.20	254.78	19.63	3.9	2.68 x 10^5^	2.90 × 10^−5^	0.108 ± 0.009
Fab-PAS#1(400)	82.5	451	14.38	870.31	5.75	10.7	1.48 × 10^5^	2.51 × 10^−5^	0.170 ± 0.017
Fab-PAS#1(600)	99.0	691	28.19	1751.36	2.85	21.0	1.08 × 10^5^	2.63 × 10^−5^	0.244 ± 0.015
Fab-2xPAS#1(200)	82.7	449	37.22	2915.36	1.72	27.8	1.22 × 10^5^	4.48 × 10^−5^	0.367 ± 0.020
Fab-ABD	53.3	40.1	28.92	2376.35	2.10	21.6	–	–	–
Fab-PEG(20)_2_	88.1	478	35.33	2726.88	1.83	26.4	1.63 × 10^5^	3.22 × 10^−5^	0.198 ± 0.016
IFN	21.0	23.7	0.54	12.62	396.25	1	3.32 × 10^6^	7.89 × 10^−3^	2.37 ± 0.02
PAS#1(200)-IFN	37.4	190	2.64	111.05	45.03	4.9	1.28 × 10^6^	11.8 × 10^−3^	9.25 ± 0.06
PAS#1(400)-IFN	54.0	416	7.90	451.52	11.07	14.6	1.47 × 10^6^	13.3 × 10^−3^	9.01 ± 0.05
PAS#1(600)-IFN	70.5	610	15.85	1171.15	4.27	29.4	1.46 × 10^6^	13.1 × 10^−3^	8.99 ± 0.06
PEG-IFN	59.2	–	–	–	–	–	0.98 × 10^6^	16.4 × 10^−3^	16.7 ± 0.12
hGH	23.6	22.5	0.047	5.67	882.13	1	9.70 × 10^5^	2.83 × 10^−5^	29.2 ± 2.0 × 10^−3^
PAS#1(600)-hGH	73.1	612	4.42	415.88	12.02	94	4.60 × 10^5^	3.58 × 10^−5^	77.8 ± 2.1 × 10^−3^

To gain further information on the conformational properties of the PAS tag, circular dichroism (CD) spectra were recorded for the IFN fusion proteins (Fig. 2F). The CD spectrum of the unfused IFN exhibited the typical features of an α-helical protein with two characteristic minima around 208 and 220 nm. However, the spectra of the corresponding PAS fusion proteins revealed an evident deviation: with increasing length of the polymer a predominant negative minimum around 200 nm arose. To visualize the spectroscopic contributions by the PAS fusion partner in greater detail, we calculated the molar difference CD spectra with respect to the unfused IFN (Fig. 2G). For all PAS fusion proteins a strong minimum at 198 nm appeared, which is clearly indicative of random coil conformation (Fändrich and Dobson, 2002). Furthermore, if considered on a molar basis, the negative amplitude at 198 nm increased proportionally to the length of the PAS tag, thus revealing that the PAS polypeptide as part of the recombinant fusion protein represents a true random chain under physiological buffer conditions. Very similar difference CD spectra were also observed for the PASylated Fab and hGH (Supplementary Fig. S3), demonstrating that the PAS random coil conformation is not influenced by the secondary structure of the biologically active fusion partner, i.e. predominantly α-helix for IFN and hGH versus β-sheet in the case of the Fab.

**Fig. 3.**
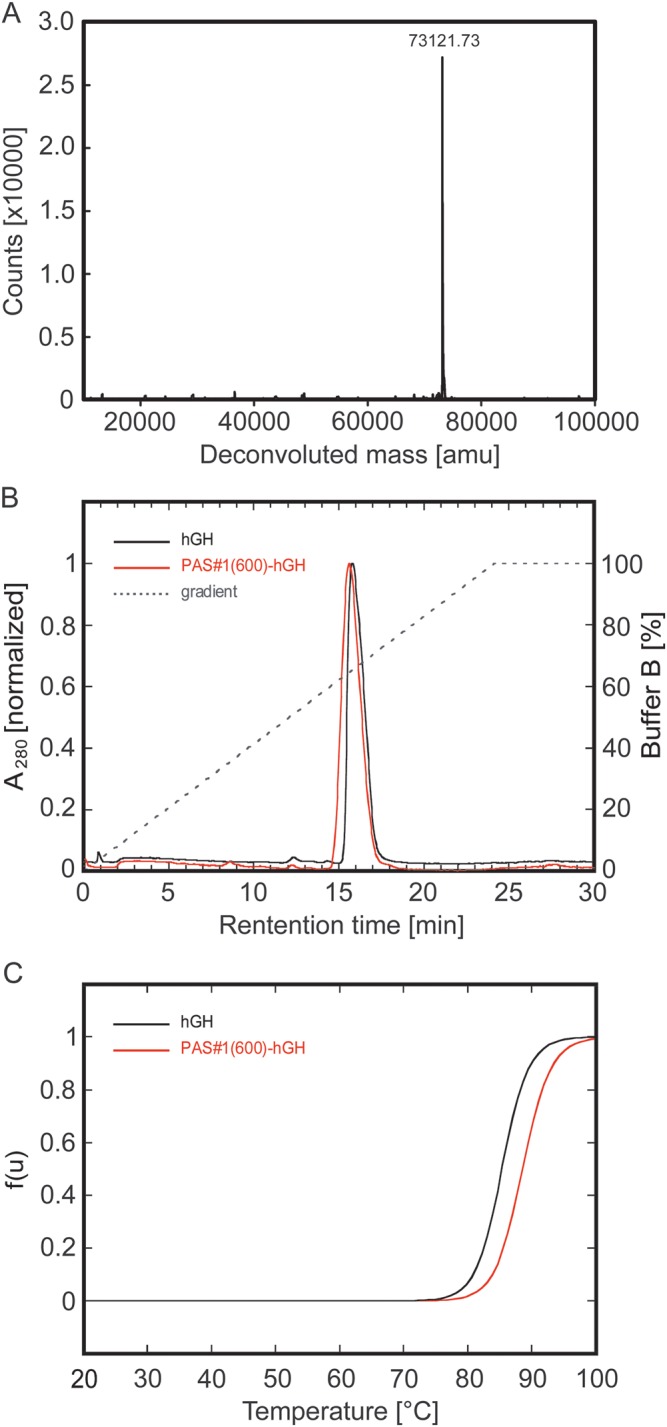
Biophysical characterization of PAS#1(600)-hGH. (**A**) Mass spectrometric analysis of PAS#1(600)-hGH by ESI-MS, confirming the calculated molecular mass of 73121.65 Da as well as precisely monodisperse composition, without any indication of prematurely terminated gene products. (**B**) Hydrophobicity analysis of PASylated hGH by reverse-phase (RP) HPLC. HIC of the original recombinant hGH and its PASylated version was performed on a Resource RPC column using an elution gradient from 2% v/v acetonitrile, 0.065% v/v TFA to 80% v/v acetonitrile, 0.05% v/v TFA. Both profiles show a single homogeneous peak with slightly earlier elution of PAS#1(600)-hGH (51.8% acetonitrile) in comparison with the corresponding unfused protein (52.3% acetonitrile). (**C**) Thermal denaturation of hGH and PAS#1(600)-hGH as determined by far UV CD measurement. Thermal unfolding of a 8.2 µM protein solution in 50 mM K_2_SO_4_, 20 mM K-P_i_ pH 7.5 was followed at a wavelength of 208 nm where maximal spectral change upon unfolding was observed and the normalized unfolded fraction, f(u), was plotted as a function of temperature. The melting temperatures (*T*_m_) of hGH and PAS#1(600)-hGH were 85.4°C and 88.6°C, respectively.

The biophysical behavior of PAS#1 fusion proteins was further investigated with regard to isoelectric and hydrophilic properties. Isoelectric focusing (IEF) experiments, carried out for PAS600-IFN (Fig. 2C) and PAS600-hGH (not shown), revealed a negligible influence on the isoelectric point (p*I*) of the biologically active protein, in line with the complete absence of charged side chains in the PAS sequence. On the other hand, hydrophobic interaction chromatography (HIC) showed a very similar, even slightly earlier elution upon decreasing solvent polarity for PAS#1(600)-hGH compared with the original protein, illustrating the low hydrophobicity of the PAS polypeptide despite the lack of strongly polar side chains (Fig. 3). Finally, we tested the influence of the PAS tag on the folding stability of the biologically active fusion partner hGH by thermal unfolding in a CD spectrometer. Both the unmodified recombinant hGH and its PASylated version showed a single unfolding transition with unchanged cooperativity (Fig. 3C). While hGH itself had a melting temperature (*T*_m_) of 85.4 °C, its PAS#1(600) fusion was even slightly more stable (*T*_m_ = 88.6 °C), demonstrating that the natively unstructured PAS tag does not negatively influence the stability of the folded protein moiety. Notably, whereas hGH showed aggregation upon thermal unfolding the cuvette stayed clear for the PASylated protein, indicating a solubilizing effect of the PAS polypeptide. Similar observations were made during thermal unfolding of the PASylated IFN (not shown).

To investigate the influence of the detailed composition of the PAS sequences (Fig. 1C) on their biophysical properties we fused the polymer PAS#5 (with 192 residues from 8 ligated 24mer cassettes) to the *N*-terminus of IFN and characterized the purified fusion protein by analytical SEC and CD spectroscopy (not shown). Again, the PAS#5 polypeptide revealed the characteristic CD minimum for a random coil, and in SEC the hydrodynamic volume of the fusion protein was increased by a factor 4.4, similar to the PAS#1(200) fusion protein. To further study the role of the Pro residues on the properties of PAS polypeptides we designed the sequence PAS#1P2 in which the number of Pro residues was reduced from 7 to 2 per 20-residue stretch (cf. Fig. 1C). The resulting 200 residue polymer (from 10 ligated 20mer cassettes) was fused both to IFN and to the 4D5 Fab fragment. Although both fusion proteins could be produced in the periplasm of *E.coli* in a soluble form they showed clearly reduced yields (ca. 30% in comparison with the corresponding PAS#1 versions). Furthermore, they exhibited a much faster electrophoretic mobility in SDS–PAGE (as shown in Supplementary Fig. S4 for the Fab fragment), indicating a more compact, less random-like conformation and/or better binding of SDS. Indeed, analytical SEC revealed a significantly smaller apparent molecular size, e.g. of 82.2 kDa for PAS#1P2(200)-IFN (cf. above). Thus, lowering the proportion of Pro results in a collapse of the conformationally expanded PAS random coil and, consequently, the Pro content appears as an important parameter for their beneficial biophysical characteristics.

### PAS fusion leads to strongly prolonged PK in mice while retaining high target-binding activity

**Fig. 4.**
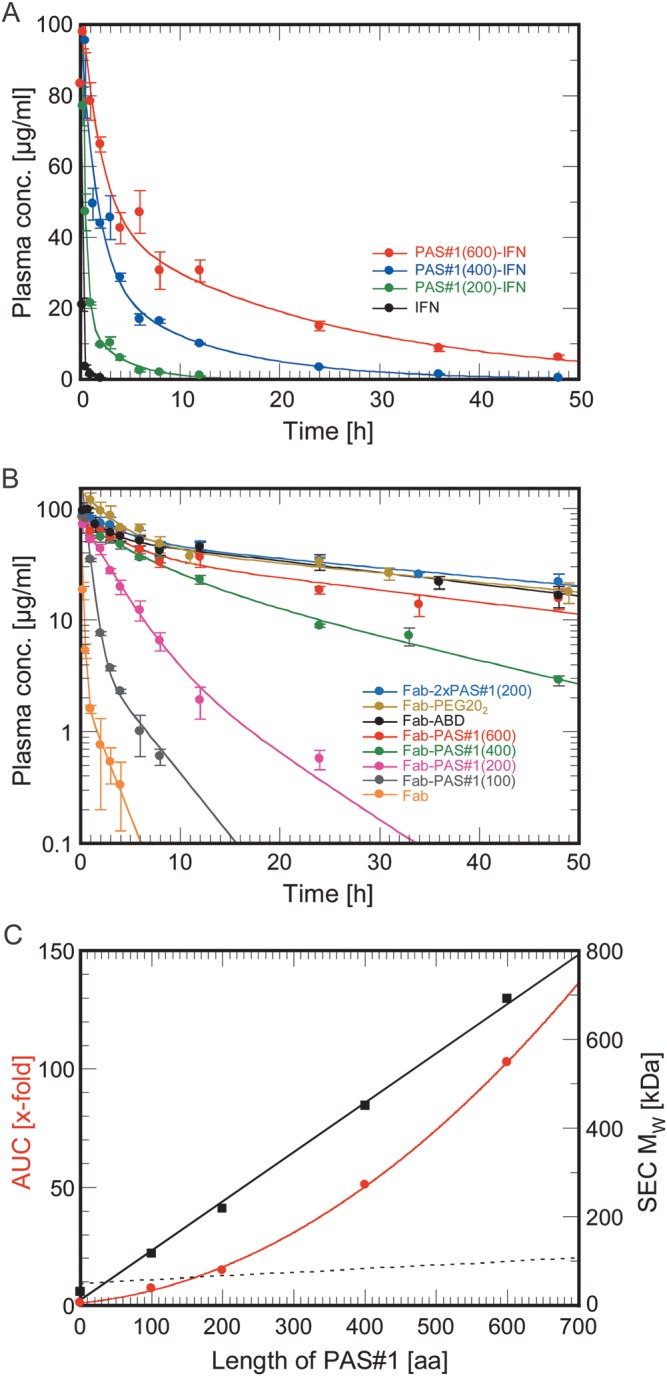
Effect of PASylation on protein PK. (**A**) PK of recombinant IFN and its PASylated versions in the blood of female BALB/c mice up to 48 h post i.v. injection at a dose of 5 mg/kg body weight (b.w.). The protein concentration in plasma was quantified in a sandwich ELISA using appropriate calibration curves and plotted against the time of sampling. Data were fitted with WinNonlin ver. 6.1 assuming a bi-exponential decay. Resulting parameters are listed in Table I. (**B**) PK of the 4D5 Fab fragment and its PASylated forms—as well as a PEGylated version and an ABD fusion (see text)—measured as in (A). Plasma concentration values were plotted in a semi-logarithmic fashion. (**C**) Plot of the AUC values (normalized to the unmodified protein, left) determined from the PK analysis in (B) and of the apparent *M*_W_ from the SEC analysis in Fig. 2D (right) against the length of the PAS sequence for the PASylated Fab fragments. For comparison, the dotted line indicates the true protein mass.

Based on the enlarged hydrodynamic volume we anticipated that conjugation of proteins with PAS polypeptides should lead to reduced clearance via kidney filtration *in vivo*, similar to coupling with PEG. Therefore, the PK of PASylated IFN and 4D5 Fab fragment was investigated in BALB/c mice and compared with the corresponding unfused proteins. After i.v. injection blood samples were taken at fixed time points and recombinant protein concentrations in plasma were determined using sandwich enzyme-linked immunosorbent assay (ELISA; Fig. 4, Table I), followed by numerical analysis with WinNonlin software.

The PAS#1-IFN fusion proteins showed bi-exponential decay, irrespective of the presence of the PAS tag, whereupon the unfused IFN exhibited the fastest excretion of all. In contrast, the values for the terminal elimination half-life of the PASylated IFN were dramatically increased depending on the length of the attached polymer, from 32 min for the unfused protein to 15.85 h for the PAS#1(600) fusion (Table I), thus demonstrating a strong prolongation effect of the PAS tag on *in vivo* PK. This was reflected by the values obtained for the clearance (decrease from 396.3 to 4.3 ml h^−1^ kg^−1^) and an almost 100-fold increase in the area under the curve (AUC), from 12.6 to 1171 h μg ml^−1^. Thus, by varying the length of the PAS polypeptide the *in vivo* half-life can be simply adjusted to the desired experimental application.

The 4D5 Fab fragment fused with the PAS#1 polypeptide at the *C*-terminus of its light chain showed a similar bi-exponential PK profile (Fig. 4B). Again, the 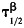 values strongly increased with growing length of the PAS sequence, culminating in a factor 21 for the PAS#1(600) fusion compared with the original Fab. Surprisingly, the duoPAS Fab, with a molecular mass almost identical to the linear Fab-PAS400 fusion, showed a terminal half-life that was even longer than the one of Fab-PAS600, with a 66% higher AUC, even though its apparent molecular size as measured by SEC was nearly 250 kDa smaller. Notably, this branched PAS fusion protein also showed a longer PK than the PEG(20)_2_ conjugate. Hence, similar to branched PEG (Jevsevar *et al.*, 2010), not only the hydrodynamic volume—which is mainly determined by the length of the polymer chain—seems to be an important parameter for kidney filtration but also the average density of the random coil. Apart from that it is noteworthy that the branched PASylated Fab fragment also showed a longer plasma half-life than the corresponding fusion protein with the *streptococcal* albumin-binding domain, which effects retarded clearance by mediating association with serum albumin (Schlapschy *et al.*, 2007).

Interestingly, when plotting the apparent molecular size measured by analytical SEC against the sequence length of the PAS#1 polypeptide, a linear relationship was observed (Fig. 4C). This was unexpected because classical polymer theory predicts a square root dependence of the average dimensions of a true random chain upon sequence length Cantor and Schimmel, 1980). When plotting the measured plasma AUC of the PASylated Fab fragments against the PAS length even an ascending slope became apparent, indicating that clearance gets increasingly sensitive to an extension of chain length—not unlike the effect of PEG conjugation described before (Mehvar, 2000). Thus, one can anticipate that still longer circulation in blood than described here may be realized via PASylation. In fact, PAS fusion proteins with up to 1000 residues were successfully prepared up to now.

To analyse the effect of the attached PAS polypeptide on the biochemical activity of the functional protein component we measured thermodynamic affinities and binding kinetics using surface plasmon resonance spectroscopy (SPR, BIAcore), both for the 4D5 Fab fragment toward its antigen, i.e. the recombinant ErbB2/Her2 ectodomain, and for IFN toward its receptor, a human IFN-α/β R2-Fc chimera (Table I; Supplementary Fig. S5). All real-time SPR traces showed typical association and dissociation phases and could be fitted to the ideal Langmuir model for bimolecular complex formation. In both cases the PASylated proteins behaved very similar to the unfused molecules, and with growing length of the PAS tag there was only a marginal increase in the dissociation constant (*K*_D_), up to a factor 2.1 for the Fab-PAS#1(600) fusion. This was mostly due to a slower rate constant of association (*k*_on_), in line with a hampered diffusion through the surface matrix of the sensorchip.

In comparison, for the PEGylated Fab fragment k_on_ was reduced to a similar extent as for the PAS#1(400) fusion. Furthermore, binding of the PASylated Fab to its native cell-bound target receptor was studied using fluorescence-activated cell sorting of the SK-BR-3 tumor cell line (Supplementary Fig. S6). In this instance a more pronounced loss in apparent affinity was observed for the PASylated Fab fragment (from 2 nM for the unmodified Fab to 15.9 nM for Fab-PAS600), possibly indicating reduced sterical accessibility of the bulky molecule to the Her2 receptor when exposed directly on the cellular plasma membrane, embedded among the glycocalyx.

In the SPR analysis of IFN, PASylation led to an increase in *K*_D_ from 2.4 nM to about 9 nM, that is by less than a factor 4. Interestingly, this decrease was essentially independent of the polymer length, indicating an effect primarily resulting from structural modification of the *N*-terminus of the cytokine rather than from the increase in hydrodynamic volume. This time, PEGylation caused a clearly larger decline in affinity by a factor 7 (see Table I).

### *In vivo* activity of PASylated hGH as an established biopharmaceutical

**Fig. 5.**
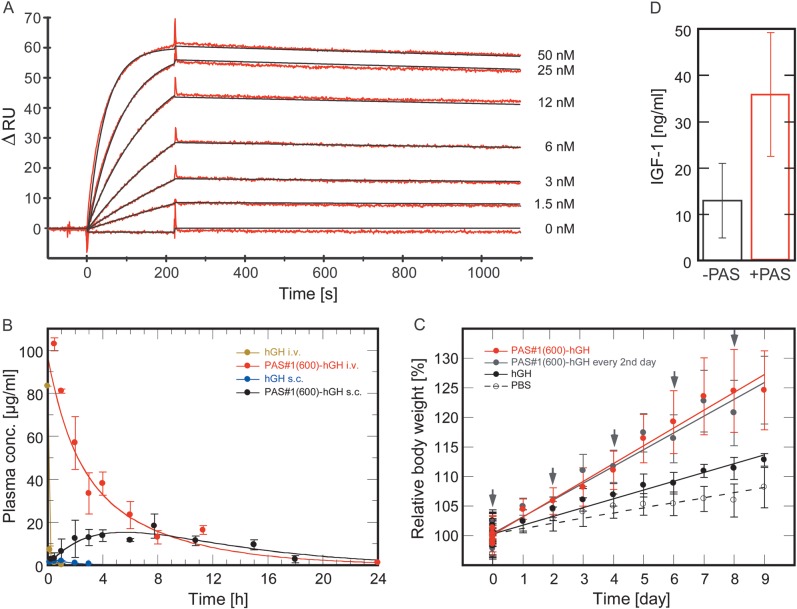
*In vitro*/*in vivo* activity and plasma half-life of PASylated hGH. (**A**) Real-time kinetic analysis of PAS#1(600)-hGH binding to the hGH receptor as Fc chimera immobilized on a Xantec CMPD sensorchip (ΔRU ≈ 200) measured on a BIAcore 2000 instrument. The signals for various hGH concentrations are depicted as red lines while curve fits according to a 1 : 1 Langmuir model are shown in black. The resulting kinetic and affinity parameters are listed in Table I. (**B**) PK of hGH and its PAS#1(600) fusion in the blood of C57BL6/J mice up to 24 h post i.v. versus s.c. injection. In the case of i.v. application, WinNonlin data analysis revealed a terminal half-life of 4.42 h for PAS#1(600)-hGH, contrasting with 0.047 h for hGH. The PK profiles for s.c. administration show distinct resorption and elimination phases with a terminal half-life of 3.72 h for PAS#1(600)-hGH. (**C**) PD study in growth-retarded homozygous (lit/lit) mice of strain C57BL/6J (age: ca. 8 weeks, weight: 10–12 g). Four groups (*N* = 7 or 8) were injected s.c. starting on Day 0 with either PBS vehicle or a fixed dose of 43 nmol/kg (b.w.) of hGH or of PAS#1(600)-hGH each day or of PAS#1(600)-hGH every second day (see arrows). Body weights were measured and normalized according to the mean b.w. for each mouse during eight days before the first injection (i.e. set to 100%). These normalized values (depicted with error bars representing the standard deviation) were averaged for each group on a daily basis and linearly fitted. The slope of the straight line represents the daily increase in b.w.: PBS: 0.86 ± 0.14%/day; hGH: 1.48 ± 0.08%/day; PAS#1(600)-hGH: 3.01 ± 0.09%/day; PAS#1(600)-hGH injected every 2nd day: 2.84 ± 0.12%/day. (**D**) Comparison of IGF-1 biomarker concentration in the plasma of mice from (C) at the end of the experiment as measured in a sandwich ELISA.

To investigate the bioactivity of a PASylated protein, hGH (somatropin) was chosen as an established model biopharmaceutical (Franklin and Geffner, 2009). Recombinant hGH as well as its fusion with a PAS#1(600) polypeptide were produced in *E.coli* and purified to homogeneity (Fig. 3A), including endotoxin depletion. According to SEC analysis the PASylated hGH showed an expanded hydrodynamic volume, larger by a factor 26 compared with the unfused protein (not shown), while CD spectroscopy indicated random coil conformation for the PAS tag with a spectral signature very similar to the PASylated IFN described above (Supplementary Fig. S3). Affinity measurements using SPR with an immobilized hGH receptor Fc chimera (Fig. 5) uncovered high binding activity with a *K*_D_ value of 77.8 ± 2.1 pM for PAS-hGH. This number was very close to that for the original recombinant protein (*K*_D_ = 29.2 ± 2.0 pM), once again demonstrating that PASylation has only a marginal effect on receptor affinity. As observed in the other instances before, the slightly higher *K*_D_ value was mainly due to a decrease in the rate constant of association (*k*_on_) whereas *k*_off_ was nearly unaffected by the PAS tag (cf. Table I).

Biochemical stability of PAS-hGH was studied *in vitro* by incubation with mouse plasma (Supplementary Fig. S7), demonstrating resistance against proteolysis for at least 2 days at 37°C. On the other hand, we investigated biodegradability of the PASylated hGH (Supplementary Fig. S8), as it is well known that PEGylated proteins can accumulate in tissues, e.g. leading to renal vacuolation (Bendele *et al.*, 1998). When the fusion protein was incubated with varying dilutions of a freshly prepared mouse kidney homogenate PAS-hGH was rapidly degraded. Consequently, in contrast to the poorly degradable chemical polymer PEG, PAS polypeptides should be easily metabolized.

The PK profile of PAS-hGH in comparison with the unfused protein was investigated both for i.v. and s.c. application, the latter to mimic clinical drug administration. In case of the i.v. bolus injection the recombinant hGH was very rapidly cleared from the blood stream (Fig. 5B) with a terminal half-life of less than 3 min, reflecting the known receptor-mediated uptake on top of renal elimination (Webster *et al.*, 2008). However, a much delayed clearance was seen with the PASylated hGH, showing a 94-fold longer terminal half-life of 4.42 h. Following s.c. application, PASylated hGH exhibited the typical PK profile according to the Bateman function (Garrett, 1994), with strong increase in plasma concentration already shortly after administration (2–4 h), indicating quick and high bioavailability. The subsequent elimination half-life was 3.72 h, similar to that of the i.v. application. Notably, AUC after s.c. injection was 50-fold larger for PAS-hGH than for the unfused protein, thus promising much increased drug exposure.

The *in vivo* bioactivity of PAS-hGH was investigated in a C57BL/6J-*Ghrhr^lit^*/J mouse model exhibiting growth retardation due to a defect in growth hormone release (Fig. 5C). The dwarfism of these ‘little’ mice can be reversed by s.c. administration of hGH (Bellini and Bartolini, 1993). To assess the normal (background) growth rate, one group of the homozygous female mice received a daily dose of buffer vehicle (phosphate-buffered saline; PBS), which led to a body weight gain of 0.86% per day. A second and third group received a daily dose of 43 nmol/kg (b.w.) of unfused hGH or of PAS#1(600)-hGH, respectively, which resulted in an additional daily weight increase of 0.62 and of 2.15%. Thus, PAS#1(600)-hGH had a more than 3-fold stronger growth-promoting effect than the original hGH. Furthermore, a fourth group received the same injection of PAS#1(600)-hGH only every second day. Remarkably, this led to a net growth rate (1.98%/day) nearly identical to that upon daily dosing, which confirms the prolonged action of the PASylated protein drug.

In all groups tested the growth effect remained essentially constant for at least 10 days and the animals showed normal behavior, food intake and drinking, resulting in a body weight gain by >25% for PAS-hGH at the end of the experiment, without any signs of drug toxicity. These findings demonstrate that PASylation can boost bioactivity of hGH in an established animal model resulting in an at least 6-fold higher PD effect—based on the average molar dose—hence allowing lower dosing at longer intervals.

The enhanced bioactivity of PAS-hGH was further confirmed by measuring the insulin-like growth factor (IGF)-1 level, which is upregulated upon stimulation via hGH in a concentration-dependent manner and serves as biomarker for drug activity (Salvatori, 2004), at the end of the experiment. In fact, PASylated hGH led to a 2.8-fold higher IGF-1 plasma concentration compared with the mice treated with hGH alone (Fig. 5D). Furthermore, organs taken from mice treated daily with PAS#1(600)-hGH showed no histological changes in kidney, liver and spleen (Supplementary Fig. S9), which supports the good tolerability of the PASylated protein. Finally, plasma taken from the mice treated daily with PAS-hGH or with hGH was tested for anti-drug antibodies (ADAs). IgG reactive against the hGH moiety was detectable on a western blot (Supplementary Fig. S10), in accordance with the known immunogenicity of hGH in mice (Lee *et al.*, 1997). However, there was no cross-reactivity with several unrelated proteins fused with the same PAS#1 sequence, indicating that the PAS polypeptide itself is not immunogenic. This finding was also supported in a mouse immunization and epitope analysis conducted with the PASylated IFN (Supplementary Figs S11–13).

## Discussion

We propose a new technology, dubbed PASylation, that enables the preparation of biologically and/or pharmaceutically functional proteins furnished with prolonged *in vivo* activity. PASylation mimics the advantageous biophysical properties of the chemical polymer PEG, especially the expanded hydrodynamic volume that provokes slow down of kidney filtration. In contrast, fusion with the biological PAS polypeptide(s) avoids obvious drawbacks of PEGylation, namely the effort for chemical conjugation with the costly protein component as well as isolation of functional product, the inherent polydispersity of the polymer and the risk of organ accumulation due to cellular uptake without an efficient route for metabolization.

The biophysical evidence available, in particular from SEC and CD experiments, clearly indicates a random chain behavior of PAS polypeptides that is responsible for their PEG-like characteristics in solution and, consequently, for the retarded renal clearance of their conjugates. Obviously, the dominating molecular contribution of the polar peptide backbone groups, together with the hydroxyl groups of Ser residues, in relation to the very small hydrocarbon side chains of the PAS amino acids cause the strongly hydrophilic properties—despite the complete lack of charges (as in PEG).

In fact, the molar ratio between apolar hydrocarbon and polar hydrogen bond donor/acceptor atomic groups, i.e. CH_n_:(NH/CO/OH), for the PAS#1 sequence is 54:43 = 1.25 whereas the corresponding value is significantly larger for PEG, with a CH_2_:O ratio of 2. Hence, as long as exposed to the solvent as part of the natively unfolded chain the polar peptide groups ensure a strongly hydrophilic character of the PAS polymer. Furthermore, the abrupt change in the random coil structure when lowering the proportion of Pro residues within a PAS sequence underlines the role of this imino acid for the beneficial properties, probably both due to its nature as secondary structure breaker and to its *N*-terminal *cis*/*trans* isomerism that increases the backbone entropy of the random chain. Apart from that, the exact sequence of the Pro, Ala and Ser amino acids seems to play a minor role. Indeed, no screening effort for the optimization of PAS sequences was necessary, once the fundamental principle had been uncovered.

All observations so far indicate a rather inert biochemical behavior of the PAS polypeptides. Generally, there is no detectable association tendency in solution, whereas a PAS tag seems to solubilize its fusion partner to a certain extent. Conversely, there is no shielding effect as sometimes described for PEG (Jevsevar *et al.*, 2010) and also for other amino acid polymer sequences (Schellenberger *et al.*, 2009); instead, PASylated proteins retain their target- or receptor-binding activities in an almost quantitative manner. Except for the rather inert alcohol group of Ser, there are no chemically reactive side chains present in the PAS polypeptides, which results in strong resistance against post-translational modification, especially hydrolysis and air oxidation. The high grade of definition of all PAS fusion proteins prepared up to now is reflected by the monodisperse mass spectra and the plain IEF profiles and should constitute a major advantage for bioprocess development.

Furthermore, PAS sequences are well compatible with the cellular secretion machinery—not only for *E.coli* but also for eukaryotic host cells such as *Pichia pastoris* and CHO or HEK cell lines (to be published)—which does not only facilitate the formation of disulfide bridges that are present in the majority of therapeutic proteins but also directly leads to a mature *N*-terminus (after intracellular processing by signal peptidase). Also, the inert and natively unfolded constitution make PAS polypeptides poor antigens for antibodies, beside their inherent lack of T-cell epitopes, which was clearly demonstrated by the absence of immune reactivity toward the PAS moiety both in animal immunization and repeated dose administration experiments.

Fab fragments with their pair of *C*-termini remote from the combining site have provided a nice model system in order to generate quasi-branched PAS tags, similarly to the branched PEG reagents that are nowadays in use for chemical conjugation (Jevsevar *et al.*, 2010). Interestingly, the branching led to a significantly stronger PK-extending effect than for a linear PAS chain of the same length. This phenomenon has been repeatedly observed in our laboratory for other proteins of interest. Of course, PAS polypeptides can also be linked to both *N*- and *C*-termini of the same protein component (provided there is no prohibitory sterical interference with target association) and they can even serve as linkers between two biochemically active proteins to generate bispecific reagents with prolonged plasma half-life.

Generally, due to the laws of allometric scaling (Mahmood, 2005) the plasma half-life of typically sized proteins subject to biomedical research—including antibody fragments, growth factors, cytokines, receptor fragments, enzymes or even bioactive peptides—in small laboratory animals like mice or rats is extremely short, usually in the range of minutes. This phenomenon is often underestimated when designing animal experiments such that overly high doses are required in order to study *in vivo* activity, which on the contrary can lead to undesired side effects.

PASylation provides a simple and inexpensive approach to researchers familiar with recombinant protein preparation in order to equip their biomolecule of interest with a satisfactory and even tunable circulation half-life. The resulting fusion protein is highly homogeneous both with regard to molecular size and to the site of PAS attachment, contrasting with most of the PEGylation techniques available to date. Moreover, upon scaling up no costly reagents or additional bioprocessing steps are needed; consequently, PASylation also offers prospects for biopharmaceutical drug development, going beyond preclinical research.

A few other structurally disordered and/or low complexity amino acid biopolymers have been proposed before in order to tailor PK properties, in particular the Gly-rich homo amino acid polymers (Schlapschy *et al.*, 2007) and the PESTAG sequences (dubbed XTEN, Schellenberger *et al.*, 2009). While the former suffer from limited solubility and an insufficient hydrodynamic volume effect the latter contain a large proportion of Glu residues, leading to high overall anionic charge and a very low p*I* value for the fusion protein—which can affect tissue distribution and cell surface receptor affinity. Nevertheless, XTEN fusion proteins have already been subject to phase I clinical trials (Schellenberger *et al.*, 2009; Cleland *et al.*, 2012), thus indicating safety and tolerability of unstructured polypeptide fusion technologies.

As we have demonstrated here, PASylation offers unique advantages, that is, surprisingly similar biophysical behavior compared with PEGylation, strong and tunable PK extending effects and conservation of high target-binding activity. Finally, this technology allows efficient secretion in microbial as well as eukaryotic host organisms due to the biochemically inert, uncharged, yet hydrophilic nature of the PAS polypeptides.

## Materials and methods

### Gene synthesis for the PAS amino acid sequences

Recombinant DNA manipulation was performed using general procedures (Sambrook *et al.*, 2001). Gene fragments encoding building blocks of 20 or 24 amino acids were obtained by hybridization of two complementary oligodeoxynucleotides as shown in Fig. 1C and ligated in a directed manner via their mutually compatible but non-palindromic sticky ends according to a previously described strategy (Schlapschy *et al.*, 2007). The synthetic gene fragments resulting from concatamer formation in the presence of a limiting amount of DNA ligase—e.g. 300 or 600 bp for a PAS#1(100) or PAS#1(200) cassette, respectively—were isolated by excising the DNA band with desired length from the ladder of ligation products after electrophoretic separation on a preparative agarose gel. These DNA fragments were initially cloned on an intermediate plasmid based on pASK75 (Skerra, 1994), which carries two *Sap*I restriction sites (5′-GCTCTTCN′NNN) in reverse orientation, leading to the same pair of sticky ends with 5′-GCC/5′-GGC overhangs. This vector (pASK-2xSapI) was cut with *Sap*I, dephosphorylated with shrimp alkaline phosphatase (USB, Cleveland, OH, USA), and ligated with the synthetic DNA fragment. After transformation of *E.coli* XL1-Blue (Bullock *et al.*, 1987), plasmids were prepared and the cloned inserts were characterized by restriction analysis and automated double-stranded DNA sequencing (ABIPrism 310 Genetic Analyzer, Applied Biosystems, Weiterstadt, Germany) using suitable flanking primers. From the resulting vector, the PAS-encoding DNA cassette could be excised again via *Sap*I digest (this time cutting on both sides of the insert) and used for subcloning as described below.

### Construction of expression plasmids for the production of Fab fragments as fusion with different PAS sequences

Starting with the previously described expression plasmid pASK88-4D5 (Schlapschy *et al.*, 2007), the *Strep*-tag II (Schmidt and Skerra, 2007) was fused to the *C*-terminus of the light chain using PCR mutagenesis, resulting in pASK88-4D5-HC-His/LC-StrepII (HC: heavy chain; LC: light chain). Then, a *Sap*I restriction site was introduced at the 3′-end of the human C_κ_ gene directly upstream of the *Strep*-tag II, generating upon cleavage a sticky end that corresponds to a GCC (Ala) codon as above. The various PAS cassettes cloned before on pASK-2xSapI were excised via restriction digest with *SapI* and ligated with the likewise cut plasmid pASK88-4D5-HC-His/LC-StrepII. To generate longer PAS-encoding inserts, an excess of the PAS DNA cassette was used for ligation, thus allowing multiple insertion in a head to tail fashion. Alternatively, a plasmid carrying already one insert was linearized again using the *Sap*I site at its upstream end and subjected to ligation with a new PAS DNA cassette. The plasmids were named pASK88-4D5-HC-His/LC-PAS#X(aa)-StrepII with X denoting the PAS version and aa indicating the length of the inserted amino acid polymer (cf. Fig. 1; SII: *Strep*-tag II).

For construction of pASK88-4D5-HC-PAS#1(200)-His/LC-PAS#1(200)-StrepII, first a *Sap*I restriction site was introduced into pASK88-4D5 at the 3′-end of the human C_H_1 gene directly upstream of the His_6_-tag. Then, the cloned PAS#1(200) gene cassette was inserted via this restriction site as before. Finally, the gene cassette for the entire Ig light chain was substituted via the *Nco*I and *Hin*dIII restriction sites with the one carrying a *C*-terminal PAS#1(200) sequence from pASK88-4D5-HC-His/LC-PAS#1(200)-StrepII.

The cDNA for human IFNα2b was amplified from the plasmid IRAMp995M1713Q (Deutsches Ressourcenzentrum für Genomforschung, RZPD, Berlin, Germany) using the primers 5′-TCTGTGGGCGCCAGCTCTTCTGCCTGTGATCTGCCTCAAACCCAC and 5′-GAACCAAAGCTTATTCCTTACTTCTTAAAC. The first primer contained a *Kas*I restriction site at the 5′-end, followed by a *Sap*I recognition site (both underlined), whereas the second primer contained a *Hin*dIII site (underlined). The PCR product was purified and digested with *Kas*I and *Hin*dIII and ligated with the vector pASK-IBA4 (IBA, Göttingen, Germany) which had been cut likewise. The cDNA for hGH was amplified from the plasmid IRATp970A09116D (RZPD) using the primers 5′-CCGCTAGCCATCACCACCATCACCATGGCGCCAGCTCTTCTGCCTTCCCAACCATTCCCTTATCC and 5′-GCCACC AAGCTTAGAAGCCACAGCTGCCC. The first primer encoded an *N*-terminal His_6_-tag and contained an *Nhe*I restriction site at the 5′-end, followed by *Kas*I and *Sap*I recognition sites (all underlined), whereas the second primer contained a *Hin*dIII site (underlined). The PCR product was purified, digested with *Nhe*I and *Hin*dIII, and ligated with the vector pASK75 (Skerra, 1994) which had been cut likewise. After transformation of *E.coli* XL1-Blue, plasmids were prepared and the sequences of the cloned inserts were confirmed by restriction analysis and double-stranded DNA sequencing. The plasmids coding for IFN and hGH as fusions with an *N*-terminal *Strep*-tag II or His_6_-tag, respectively, were designated pASK-IBA4-IFN and pASK75-His_6_-hGH.

For the construction of corresponding expression plasmids encoding IFN or hGH as fusion proteins with PAS#1 sequences of different lengths, pASK-IBA4-IFN or pASK75-His_6_-hGH were cut with *Sap*I, dephosphorylated with shrimp alkaline phosphatase, and ligated with an excess of the cloned PAS#1(200) gene cassette from above. After transformation of *E.coli* JM83 (Yanisch-Perron *et al.*, 1985), plasmids were prepared and the presence and size of the inserts were confirmed by restriction analysis. The resulting plasmids were designated pASK-IBA4-PAS#1(200/400/600)-IFN and pASK75-PAS#1(200/400/600)-hGH, respectively.

### Recombinant protein production and purification

All recombinant proteins were produced in *E.coli* KS272 (Meerman and Georgiou, 1994) harboring the corresponding expression plasmid and, if useful, the helper plasmid pTUM4 (Schlapschy *et al.*, 2006). Bacteria were cultivated either in shake flasks containing 2 l LB medium (Sambrook *et al.*, 2001) supplemented with 100 mg/l ampicillin, 30 mg/l chloramphenicol (for pTUM4), 1 g/l proline and 5 g/l glucose or, alternatively, in a 4- or 8-l bench-top fermenter with a synthetic glucose mineral salt medium supplemented with 100 mg/l ampicillin, 30 mg/l chloramphenicol and 1 g/l proline according to a published procedure (Schiweck and Skerra, 1995). In the shake flask, recombinant gene expression was induced with 200 μg/l anhydrotetracycline (aTc) (Skerra, 1994) at OD_550_ = 0.5 for up to 3 h at 22°C. During fermenter production, induction was achieved by addition of 500 μg/l aTc as soon as the culture reached OD_550_ = 20 for a period of up to 2.5 h. Immediately thereafter, the cells were harvested by centrifugation and a periplasmic extract was prepared in the presence of 500 mM sucrose, 1 mM EDTA, 100 mM Tris/HCl pH 8.0 supplemented with lysozyme as appropriate.

The Fab fragments and hGH were purified from the periplasmic extract via the His_6_-tag using a Ni^2+^ charged HisTrap HP IMAC column (GE Healthcare, Uppsala, Sweden) (Schiweck and Skerra, 1995). To further purify the PASylated Fab fragments or to isolate IFN from the periplasmic extract, *Strep*-Tactin affinity chromatography (Schmidt and Skerra, 2007) was employed. All proteins were finally polished by SEC on Superdex 75 or 200 pg HiLoad 16/60 columns (GE Healthcare) in PBS (4 mM KH_2_PO_4_, 16 mM Na_2_HPO_4_, 115 mM NaCl).

Purified proteins were concentrated by ultrafiltration using Amicon Ultra centrifugal filter units (10 000 or 30 000 MWCO; 4 ml or 15 ml; Millipore, Billerica, MA, USA) to about 1.0 mg/ml. For endotoxin removal proteins were applied to anion exchange chromatography (Q Sepharose Fast Flow, GE Healthcare), followed by an EndoTrap Red depletion step (Hyglos, Regensburg, Germany). Typical endotoxin contents were below 10 EU/mg of protein as measured with an Endosafe-PTS system using cartridges with 0.1–10 EU/ml sensitivity (Charles River Laboratories, Wilmington, MA, USA).

SDS–PAGE was performed using a high molarity Tris buffer system (Fling and Gregerson, 1986) followed by Coomassie brilliant blue staining. Protein concentrations were determined according to the absorption at 280 nm using calculated extinction coefficients (Gill and von Hippel, 1989) of 68 290 M^−1^ cm^−1^ for the original 4D5 Fab fragment, 72 130 M^−1^ cm^−1^ for its ABD fusion, 73 980 M^−1^ cm^−1^ for its fusion with the different PAS sequences (which themselves show no ultraviolet absorption at this wavelength), 16 050 M^−1^ cm^−1^ for hGH and its fusions and 23 590 M^−1^ cm^−1^ for IFN and its fusions. Reported yields of purified protein [mg L^−1^ OD^−1^] were normalized to 1 l of bacterial culture and OD_550_ at harvest of 1.0.

Analytical SEC was performed on a Superdex 200 HR 10/300 GL column (GE Healthcare) at a flow rate of 0.5 ml/min using an Äkta Purifier 10 system (GE Healthcare) with PBS as running buffer. The purified proteins (250 μl) were applied at a concentration of 0.25 mg/ml. ESI-MS was recorded using an Agilent (Santa Clara, CA, USA) 6210 time-of-flight LC/MS instrument.

### CD spectroscopy

Secondary structure was analysed using a J-810 spectropolarimeter (Jasco, Groß-Umstadt, Germany) equipped with a quartz cuvette 106-QS (0.1 mm path length; Hellma, Muehllheim, Germany). Spectra were recorded at room temperature from 190 to 250 nm by accumulating 32 runs (bandwidth 1 nm, scan speed 100 nm/min, response 4 s) using 3–20 μM protein solutions in 50 mM K_2_SO_4_, 20 mM K-P_i_ pH 7.5. After correction for buffer blanks, spectra were smoothed using the instrument software and the molar ellipticity Θ_M_ was calculated according to the equation Θ_M_ = Θ_obs_/(*cd*), wherein Θ_obs_ denotes the measured ellipticity, c the protein concentration [mol/l], and *d* the path length of the quartz cuvette [cm]. The normalized data were plotted—if applicable, after mutual subtraction—against the wavelength using KaleidaGraph software (Synergy Software, Reading, PA, USA).

For thermal unfolding, solutions of hGH and PAS#1(600)-hGH at a protein concentration of 8.2 µM in 50 mM K_2_SO_4_, 20 mM K-P_i_ pH 7.5 were applied in a thermostated 1 mm path length cuvette sealed with a teflon lid. The sample was heated at a constant temperature gradient of 60 K/h from 20°C to 100°C. Data were collected each 0.2 K step at a wavelength of 208 nm where maximal spectral change upon unfolding was observed. Data were fitted by non-linear least-squares regression using KaleidaGraph and an equation for a two-state model of the unfolding transition as previously described (Schlehuber and Skerra, 2002). Using the parameters from the corresponding curve fit, the normalized unfolded fraction, f(u), was plotted as a function of temperature.

### PK animal experiments

For PK studies, female BALB/c mice (18–20 g) from Charles River Laboratories were injected i.v. with the unfused 4D5 Fab fragment, its PAS#1(100/200/400/600) or ABD fusions or the PEG(20)_2_ conjugate, and with the unfused IFN or its PAS#1(200/400/600) fusions. In the case of hGH and its PAS#1(600) fusion female C57BL/6 mice (18–20 g) (Jackson Laboratory, Bar Harbor, ME, USA) were used for i.v. and s.c. injection, in line with the genetic background of the ‘little’ mice used in the PD study described further below.

All mice per group (*N* = 3) simultaneously received a dose of 5 mg protein/kg (b.w.) in PBS, typically 100 µl of a 1 mg/ml protein solution for a mouse with 20 g b.w. Blood samples were taken after 15 min, 30 min, 1 h, 2 h, 3 h, 4 h, 6 h, 8 h, 12 h, 24 h, 36 h and 48 h from the tail vein (group I: 15 min, 2 h, 6 h and 24 h; group II: 30 min, 3 h, 8 h and 36 h; group III: 1, 4, 12 and 48 h). The plasma was prepared by centrifugation at 4°C and 14 000 rpm for 20 min and stored at −20°C.

To determine the plasma half-life of the 4D5 Fab fragment, IFN or hGH as well as their PASylated versions, the concentration values, determined from ELISA measurements (see below), were plotted against time post injection and numerically fitted using WinNonlin version 6.1 software (Pharsight, St Louis, MO, USA). Bi-exponential decay or a two-compartment system (in line with the Bateman function) were assumed in the case of i.v. bolus or s.c. injections, respectively. Data (including standard deviations) and curve fits were finally plotted with KaleidaGraph.

### PD study of *in vivo* activity for PASylated hGH

*In vivo* bioactivity of PASylated hGH in comparison with hGH was studied in growth retarded dwarf mice as an established animal model (Bellini and Bartolini, 1993). To this end, female ‘little’ mice (C57BL/6J lit/lit; Jackson Laboratory) at an age of around 8 weeks and with an average body weight of 10–12 g were used. All animals were kept in clear plastic cages in an air-conditioned animal house. The temperature was maintained at 22°C and lighting was regulated on a 12 h light, 12 h dark schedule. Mice were divided into four groups with *N* = 7–8 animals. Groups I and II received for up to 9 days a daily s.c. dose of 43 nmol/kg (b.w.) in 50 µl PBS of hGH (=1.0 mg/kg (b.w.)) or PAS#1(600)-hGH (=3.12 mg/kg (b.w.)), respectively. Group III received an s.c. vehicle injection of 50 µl PBS whereas group IV was injected with PAS#1(600)-hGH with the same dosage as group II but only every second day.

Animal body weight was measured daily on a balance for 8 days (Day −7 to Day 0) prior to the first sample injection (on Day 0) and then throughout the entire period of the assay (Days 1–9). For each group the mean value of the body weight was determined on each day. Then, the accumulated mean value for the first 8 days (no injections, including Day 0) was calculated and used as starting point in order to calculate the relative increase in average body weight for each day during the treatment period. These values were plotted against time and linearly fitted to deduce the growth rate using KaleidaGraph.

### ELISA for quantification of recombinant proteins in animal plasma

In the case of the 4D5 Fab fragment and its PASylated versions, 96-well microtiter plates (12 × 8 well ELISA strips with high binding capacity; NUNC, Roskilde, Denmark) were coated for 1 h with 50 μl of the recombinant Her2/ErbB2 ectodomain (kindly provided by Tim Adams, CSIRO, Parkville, Australia) at a concentration of 10 μg/ml in 50 mM NaHCO_3_ pH 9.6 at room temperature. For quantification of human IFN and its PASylated versions microtiter plates were coated with 100 μl of a 5 μg/ml solution of the mouse anti-human IFNα2b antibody 9D3 (Abcam, Cambridge, UK) in the same buffer. For quantification of hGH and its PASylated versions the microtiter plates were coated with 50 µl of a 0.8 µg/ml solution of the mouse anti-hGH antibody GhG2 (Abcam).

After that the wells were blocked with 200 µl 3% (w/v) bovine serum albumin in PBS containing 0.1% Tween 20 (PBS/T) for 1 h and washed three times with PBS/T. The plasma samples were applied in dilution series in PBS/T supplemented with up to 0.5% (v/v) mouse plasma from an untreated animal (to maintain constant content of sample matrix) and incubated for 1 h. The wells were then washed three times with PBS/T and incubated for 1 h either with 50 μl of a 1 : 1000 dilution in PBS/T of anti-human Cκ-light chain IgG alkaline phosphatase conjugate (DAKO, Glostrup, Denmark), to detect the Fab, or with 50 μl of a 1 : 1000 diluted solution of an unrelated mouse anti-human IFNα2b antibody horseradish peroxidase (HRP)-conjugate (4E10-HRP; Abcam) or with 50 µl of a 1 : 1000 dilution of an unrelated mouse anti-hGH antibody HRP-conjugate (GhB9-HRP; Abcam).

After washing twice with PBS/T and twice with PBS the enzymatic activity was detected using *p*-nitrophenyl phosphate or 2,2′-azino-bis(3-ethylbenzothiazoline-6-sulphonic acid) diammonium salt (ABTS) as chromogenic substrates for the phosphatase and peroxidase reporter enzymes, respectively. After 15 min at 25°C the absorbance at 405 nm was measured using a SpectraMax 250 microtiter plate reader (Molecular Devices, Sunnyvale, CA, USA) and concentrations of the recombinant proteins in the plasma samples were quantified by comparison with standard curves that had been determined for dilution series of the corresponding purified recombinant proteins at defined concentrations in PBS/T containing 0.5% (v/v) mouse plasma from untreated animals.

### IGF-1 ELISA

To test the effect of hGH on an established biomarker (Yakar *et al.*, 2002) at the end of the mouse PD study, IGF-1 titers in the ‘little’ mice were measured using a commercial Quantakine sandwich ELISA kit (R&D Systems, Minneapolis, MN, USA). Plasma samples taken on Day 9 from 7 or 8 mice treated daily with hGH or PAS#1(600)-hGH, respectively, were first diluted 1 : 50 with calibrator diluent. A concentration series with three further dilutions (1 : 100, 1 : 200, 1 : 400) was then prepared and the IGF-1 concentrations were determined by ELISA using a standard curve with mouse IGF-1 of known concentration (provided with the kit). Mean concentrations and standard deviations from these measurements were first calculated for each mouse and then for the two groups. A heteroskedastic T-test using Excel software (Microsoft, Redmond, WA, USA) revealed a *P* value of 0.0016 (cf. Fig. 5D).

### BIAcore real-time affinity measurements

Surface plasmon resonance spectroscopy was performed on a BIAcore 2000 or BIAcore X Instrument (BIAcore, Uppsala, Sweden). To measure antigen affinity of the 4D5 Fab fragment and its PASylated versions the recombinant Her2/ErbB2 ectodomain (100 µg/ml in 10 mM Na-acetate pH 4.5) was immobilized on a CMD200L sensorchip (Xantec, Düsseldorf, Germany) using an amine coupling kit (GE Healthcare), resulting in a surface density of around 540 resonance units (ΔRU). In the case of IFN and hGH a CMPD chip (Xantec) was used and the corresponding receptor ectodomains were applied as Fc fusion proteins (R&D Systems) and immobilized via an amine-coupled anti-Fc antibody (Jackson Immuno Research, West Grove, UK), typically leading to ΔRU of 200–250. The Fab fragment, IFN and hGH were injected in appropriate concentration series using PBS containing 0.005% (v/v) Tween 20 as running buffer. Complex formation was observed at a continuous flow rate of 25 µl/min and the kinetic parameters were determined by fitting data to a Langmuir binding model for bimolecular complex formation using BIAevaluation software version 4.1 (BIAcore). The sensorgrams were corrected by double subtraction of the corresponding signals measured for the in-line control blank channel and an averaged baseline determined from several buffer blank injections (Myszka, 1999). Chip regeneration was achieved with 10 mM glycine/HCl pH 2.2, 500 mM NaCl in the case of the Fab fragment and with 10 mM glycine/HCl pH 2.7 in the case of IFN and hGH.

### PEGylation of the 4D5 Fab fragment

To prepare a site-specifically PEGylated 4D5 Fab fragment a version without interchain disulfide bridge was used. Therefore, the Cys residue at the *C*-terminus of the C_H_1 domain was deleted such that the free singular Cys residue at the *C*-terminus of C_κ_ was available for coupling with a maleimide conjugate of branched PEG(20)_2_. The Fab fragment was produced in *E.coli* via periplasmic secretion in the 2-l shake flask as described above and its free thiol group was first activated by mild reduction with 10 mM 2-mercaptoethanol for 30 min at 30°C. After buffer exchange via gel filtration on a PD-10 desalting column (GE Healthcare), equilibrated with nitrogen-saturated 100 mM Tris-acetate pH 5 containing 5 mM EDTA, 41.7 nmol of the Fab fragment in a volume of 1.9 ml was mixed with the 3-fold molar amount of PEG(20)_2_ (Sunbright GL2-MA400; NOF, Tokyo, Japan) dissolved in 100 µl water. The coupling reaction, with a total volume of 2 ml, was started by addition of approximately 70 µl 1 M Tris base to adjust the pH to 7.5. After 1 h incubation at room temperature uncoupled Fab was removed by SEC on a Superdex S200 pg HighLoad 16/60 column. Excess PEG(20)_2_ was finally removed by IMAC on a Zn^2+^ charged IDA sepharose column (Chelating Sepharose Fast Flow; GE Healthcare). Eluate fractions were analysed by SDS–PAGE, pooled, and dialysed against PBS. After concentration by ultrafiltration using Amicon Ultra centrifugal filter devices (30 000 MWCO; 4 ml; Millipore) to about 1.5 mg/ml, endotoxin was removed by an EndoTrap Red depletion step (Hyglos), and the 1 mg/ml protein solution was stored at −20°C.

### Isoelectric focusing

IEF was performed with the multi electrophoresis chamber IEF-system (SCIE-PLAS, Cambridge, UK) according to the manufacturer's instructions using precast IsoGel agarose IEF gels (Lonza, Allendale, NJ, USA) with a pH range of 3–10. 2 µg of each protein sample was applied in 4 M urea solution in a total volume of 2 µl. The pH range 3.6–9.3 IEF mix (Sigma-Aldrich, St. Louis, MO, USA) was applied as standard. Gels were run for 20 min at 1 W, followed by 100 min at 500 V and by 100 min at 1000 V. Then, the gel was dried overnight on blotting paper (Lonza) at ambient temperature, washed in water for 5 min and again dried at 55°C. After incubation in fixing solution (36% v/v methanol, 6% w/v trichloroacetic acid, 3.6% w/v sulfosalicylic acid) for 4 h the gel was stained with 1% w/v Coomassie brilliant blue (AppliChem, Darmstadt, Germany) in 25% v/v ethanol, 9% v/v acetic acid. After destaining in 25% v/v ethanol, 9% v/v acetic acid the gel was dried and scanned. Theoretical p*I* values were calculated with the UWGCG software package (Devereux *et al.*, 1984).

### Reverse-Phase HPLC

The purified protein solution in PBS was adjusted to 2% v/v acetonitrile, 0.065% v/v trifluoroacetic acid (TFA) and 1 ml was applied to a Resource RPC 1 ml column (GE Healthcare) equilibrated with buffer A (2% v/v acetonitrile, 0.065% v/v TFA). For elution, a 0–100% gradient of buffer B (80% v/v acetonitrile, 0.05% v/v TFA) was applied over 20-bed volumes at a flow rate of 2 ml/min while monitoring protein elution at both 280 and 225 nm (PAS only absorbs in the peptide backbone region due to the absence of aromatic side chains).

## Supplementary data

Supplementary data are available at *PEDS* online.

## Funding

This work was financially supported by the Leading Edge Cluster m^4^ (grant no. 01EX1022B and 01EX1022C) funded by the Bundesministerium für Bildung und Forschung (BMBF), Germany. Funding to pay the Open Access publication charges for this article was provided by XL-protein GmbH.

## Supplementary Material

Supplementary Data
