# Supplementary material for: PASylation: a biological alternative to PEGylation for extending the plasma half-life of pharmaceutically active proteins
Source: Protein Eng Des Sel. 2013 Jun 10;26(8):489–501. doi: 10.1093/protein/gzt023 (PMC3715784; doi:10.1093/protein/gzt023)
Supplement: Supplementary Data [file supp_26_8_489__index.html]

PASylation: a biological alternative to PEGylation for extending the plasma half-life of pharmaceutically active proteins — PASylation: a biological alternative to PEGylation for extending the plasma half-life of pharmaceutically active proteins — Supplementary Data 

# PASylation: a biological alternative to PEGylation for extending the plasma half-life of pharmaceutically active proteins

## Supplementary Data

Supplementary Data

**Files in this Data Supplement:**

- Supplementary Data - Pdf file
