# Supplementary material for: PASylation: a biological alternative to PEGylation for extending the plasma half-life of pharmaceutically active proteins
Source: Protein Eng Des Sel. 2013 Jun 10;26(8):489–501. doi: 10.1093/protein/gzt023 (PMC3715784; doi:10.1093/protein/gzt023)
Supplement: Supplementary Data [file supp_gzt023_gzt023supp.pdf]

PASylation: a biological alternative to PEGylation for extending the plasma-half life of pharmaceutically active proteins

Martin Schlapschy, Uli Binder, Claudia Börger, Ina Theobald, Klaus Wachinger, Sigrid Kisling, Dirk Haller, Arne Skerra

Supporting information

Measurement of target-binding activity by FACS

To determine the affinities of the recombinant 4D5 Fab fragment and its PASylated variants towards the Her2 target receptor on living cells, a cell binding assay based on the HER2 overexpressing cell line SK-BR-3 (Kraus *et al.*, 1987) was performed. EC_{50} values of the Fab fragment and its PASylated versions were determined by competitive displacement of the unfused recombinant Fab labeled with 6-[fluorescein-5(6)-carboxamido]hexanoic acid NHS ester (Sigma-Aldrich, St. Louis, MO) at a molar ratio of 2:1. Briefly, 2 x 10^5 cells were incubated for 30 min at 4 °C with the fluorescein-labeled Fab fragment as tracer at a fixed concentration of 20 nM in the presence of the PASylated versions with varying concentrations, ranging from 20 pM to 2 µM. After washing, the fluorescence at 530 nm upon excitation at 488 nm of each sample was measured with a FACSAria cell-sorting system (BD Biosciences, Heidelberg, Germany) and data was analysed with the program FlowJo (BD). After subtraction of the signals for dead cells identified by propidium iodide staining (PI; Sigma-Aldrich), the mean fluorescence of viable cells was plotted against the logarithm of the concentration of the unlabeled PASylated Fab versions. Data was fitted to a sigmoidal equation (Kim *et al.*, 2009), resulting in EC_{50} values, from which the apparent K_D was calculated using the Cheng Prusoff equation (Cheng and Prusoff, 1973).

Stability assay of PASylated proteins in the presence of mouse serum and kidney extracts

10 µg of PAS#1(600)-hGH in PBS was incubated in pooled Balb/C mouse plasma from untreated animals (83 % v/v) at 37 °C for up to 48 h. Samples were taken at different time points, immediately diluted 1:10 with SDS-PAGE running buffer and adjusted with SDS gel loading buffer (5 x concentrated) to 2.5 % v/v 2-mercaptoethanol, followed by heating to 95 °C for 5 min. Then, the samples were subjected to SDS-PAGE (0.33 µg PAS#1(600)-hGH per lane), and a semi-dry Western blot was prepared, followed by detection with a 1:1000 dilution of anti-hGH-HRP conjugate (GhB9-HRP; Abcam, Cambridge, UK).

A kidney from a Balb/C mouse was mechanically homogenized at 4 °C in 1 ml 20 mM HEPES/NaOH, 10 mM KCl, 1.5 mM $MgCl_2$, 0.5 % v/v glycerol, pH 7.6 and the homogenate was cleared by centrifugation. 10 µg of PAS#1(600)-hGH in 15 µl PBS was mixed with 55 µl

of the kidney homogenate at varying dilution (1:5, 1:10, 1:100, 1:500, 1:1000) in PBS and incubated at 37 °C for 1 h. Samples were finally diluted 1:10 with SDS-PAGE running buffer, and a Western blot was performed as described above.

Histological analysis of mouse organs after treatment with PAS#1(600)-hGH

After completion of the mouse pharmacodynamic study, two mice from each group treated daily either with the unfused hGH or PAS#1(600)-hGH were subjected to histological analysis of kidney, liver, and spleen (mfd Diagnostics, Wendelsheim, Germany). Animals were sacrificed and the corresponding organs were isolated and stored in 10 % formalin at 4 °C. Paraffin-fixed tissue sections were prepared and stained with haematoxylin/eosin solution. Microscopic analysis was performed using an AXIO Imager A1/M1 (Carl Zeiss, Jena, Germany).

Analysis of mouse sera after PAS#1(600)-hGH treatment for immune reactivity

0.33 µg of different recombinant test proteins, all produced in *E. coli* and purified as described, were applied to lanes of the same 12 % SDS gel (reduced) and blotted on a nitrocellulose membrane. This Western blot was incubated with a 1:2500 dilution of sera from individual mice of the mouse pharmacodynamic study treated daily either with the unfused hGH or PAS#1(600)-hGH. Bound murine antibodies were detected with a 1:1000 dilution of goat anti-mouse polyvalent Ig/AP conjugate (A0162; Sigma/Aldrich).

Mouse immunization and serum analysis by ELISA and Western blotting

For immunization with PASylated IFN, 50 µg (in 50 µl PBS) of the PAS#1(200)-IFN, PAS#5(192)-IFN or unfused IFN were mixed with 150 µl 0.9 % w/v NaCl and injected intraperitoneally (*i.p.*) into each two female Balb/C mice (20 g). In the case of PAS#1(200)-IFN, also an analogous immunization experiment with Freund's adjuvans was performed. Therefore, 50 µg protein were mixed with 50 µl 0.9 % w/v NaCl as well as 100 µl complete Freund's adjuvans (Sigma-Aldrich) and injected *i.p.* Plasma samples were taken after 7, 14, and 21 days.

On day 21 the immune response was boosted (this time using incomplete Freund's adjuvans in the second immunization experiment with PAS#1(200)-IFN) and additional plasma samples were taken 2 and 7 days thereafter. These samples were analysed by ELISA for the presence of antibodies against the respective immunogen. Briefly, a Maxisorb microtitre plate (NUNC, Roskilde, Denmark) was coated overnight with 50 µl of the corresponding immunogen at a concentration of 25 µg/ml in PBS. After washing, a dilution series of each serum sample was applied and bound murine antibodies were detected with an anti-mouse polyvalent Ig/AP conjugate (A0162; Sigma-Aldrich).

For further analysis, mouse sera were tested for the presence of anti-PAS antibodies by Western blotting. To this end, a set of different test proteins (PAS#1(200)-IFN; PAS#1(200)-IL-1ra; PAS#5(192)-IFN; PAS#5(192)-IL-1ra; IFN; each 0.6 μg; not reduced) was subjected to SDS-PAGE and Western blotting, followed by incubation with appropriately diluted sera collected on day 28. Bound murine antibodies were detected with a anti-mouse polyvalent Ig/AP conjugate (A0162; Sigma-Aldrich). The PASylated versions of the interleukin receptor antagonist 1 (IL-1ra) (Holt *et al.*, 2008) or of murine leptin (Margetic *et al.*, 2002) were produced in *E. coli* in a similar manner as the IFN fusion proteins.

SPOT synthesis of immobilized peptide arrays and epitope analysis

Arrays of 24 overlapping 12mer peptides covering the entire amino acid sequence of both a PAS#1(20) cassette and a PAS#5(24) cassette – together with the *Strep*-tag II as well as the linker region N-terminally of the PAS polymer as present in the recombinant proteins used for mouse immunization – were prepared using the SPOT synthesis technique (Frank, 2002) on an automated synthesizer (MultiPep, Intavis, Germany). Briefly, the peptides were synthesized on an amino-functionalized cellulose membrane as distinct spots. A β-alanine dipeptide spacer was inserted between the C-terminus of each peptide and the membrane support. The peptide was extended stepwise by using standard fluorenylmethoxycarbonyl solid-phase peptide synthesis, followed by cleavage of the side chain protecting groups under trifluoroacetic acid conditions. Sequence files were generated with the software DIGEN (Jerini, Berlin, Germany). All peptides were N-terminally acetylated.

For an epitope peptide spot analysis, the ELISA-positive serum from above of one mouse immunized with PAS#1(200)-IFN in the presence of Freund's adjuvans and one mouse immunized with PAS#5(192)-IFN were chosen. First, the membrane was incubated in ethanol for 2 min, washed three times for 10 min with Tris-buffered saline (TBS; 50 mM Tris/HCl pH 8.0, 137 mM NaCl, 2.7 mM KCl) and blocked over night at 4 °C with 10 % w/v blocking reagent (Roche Diagnostics, Penzberg, Germany) in membrane blocking solution (MBS; 50 mM Tris/HCl pH 8.0, 137 mM NaCl, 2.7 mM KCl, 0.05 % v/v Tween-20, 1 % w/v sucrose). The membrane was washed for 10 min in MBS and incubated for 1 h at room temperature with a 1:1000 dilution of the test serum. After three washing steps for 5 min in MBS the membrane was incubated for 1 h with a 1:1000 dilution of anti-mouse polyvalent Ig/AP-conjugate (Sigma-Aldrich).

In control experiments equivalent membranes were incubated with the anti-mouse polyvalent Ig/AP conjugate only or with a 1:1000 dilution of *Strep*-Tactin/AP conjugate (IBA, Göttingen, Germany). Finally, the membrane was washed twice with TBS containing 0.05 % v/v Tween-20 and once with citrate-buffered saline (CBS; 10 mM Na-citrate pH 7.0; 137 mM NaCl, 2.7 mM KCl, 0.05 mM $MgCl_2$). For signal development the membrane was incubated with 10 ml

CBS containing 40 µl BCIP (60 mg/ml 5-bromo-4-chloro-3-indolylphosphate p-toluidine salt in dimethylformamide) and 60 µl MTT (50 mg/ml 3-[4,5-dimethylthiazol-2-yl-2,5-diphenyl-tetrazolium bromide in 70 % dimethylformamide) as chromogenic substrates (Carl Roth, Karlsruhe, Germany) for around 15 min and then washed with water, dried, and scanned.

Supporting References

Cheng, Y. and Prusoff, W.H. (1973) *Biochem Pharmacol,* **22**, 3099-3108.

Frank, R. (2002) *J Immunol Methods,* **267**, 13-26.

Holt, L.J., Basran, A., Jones, K., Chorlton, J., Jespers, L.S., Brewis, N.D. and Tomlinson, I.M. (2008) *Protein Eng Des Sel,* **21**, 283-288.

Kim, H.J., Eichinger, A. and Skerra, A. (2009) *J Am Chem Soc,* **131**, 3565-3576.

Kraus, M.H., Popescu, N.C., Amsbaugh, S.C. and King, C.R. (1987) *EMBO J,* **6**, 605-610.

Margetic, S., Gazzola, C., Pegg, G.G. and Hill, R.A. (2002) *Int J Obes Relat Metab Disord,* **26**, 1407-1433.

Supporting Figures

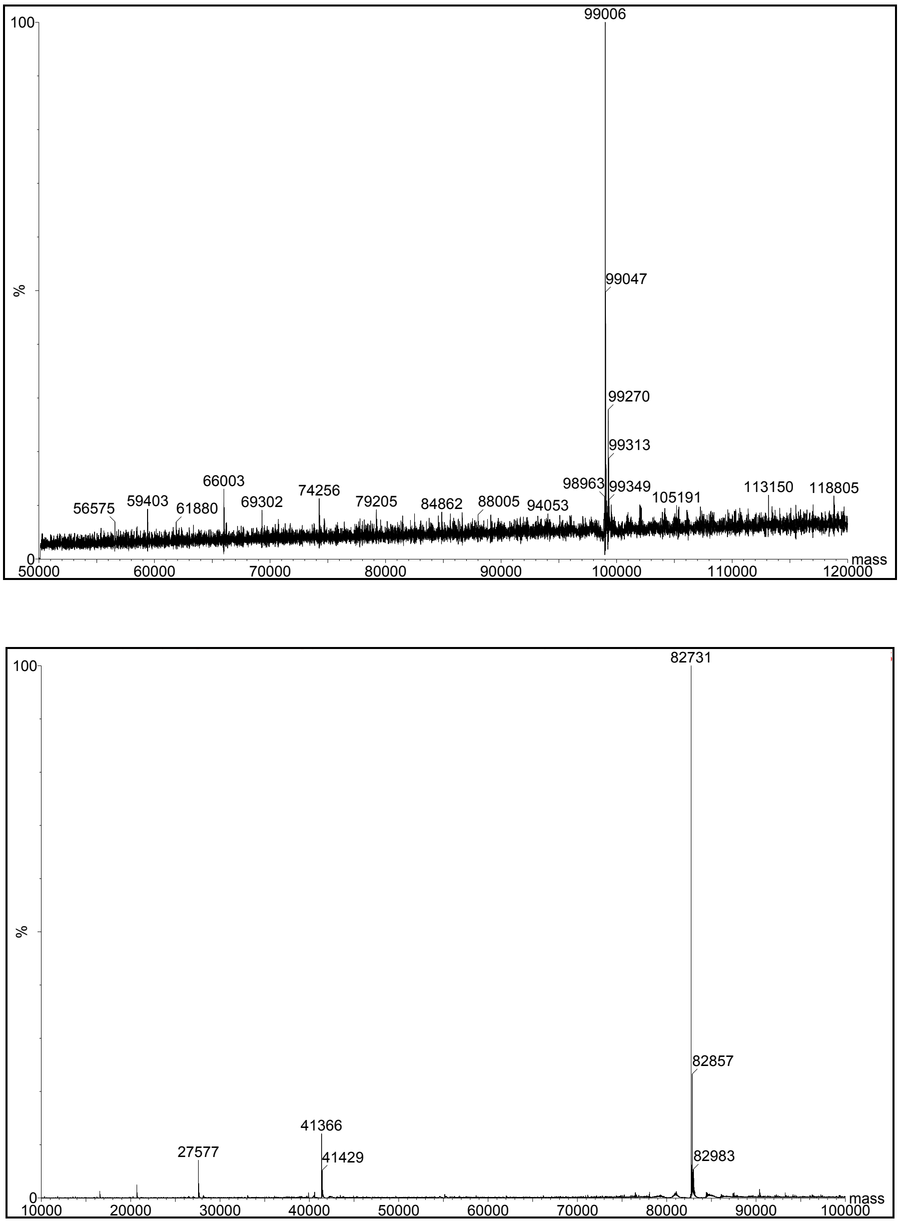

Fig. S1: ESI-MS analysis of PASylated Fab fragments.

Top: 4D5-HC-His/LC-PAS#1(600)-StrepII (dubbed Fab-PAS#1(600)) with a calculated mass of 99001 Da. Bottom: 4D5-HC-PAS#1(200)/LC-PAS#1(200)-StrepII (Fab-2xPAS#1(200)) with a calculated mass of 82728 Da. ESI-MS analysis confirms for both PASylated Fab fragments the expected molecular mass and, notably, monodisperse composition, without indication of prematurely terminated gene products. Furthermore, the PAS polymer does not hamper the correct association of Ig light and heavy chains via disulfide bridge formation.

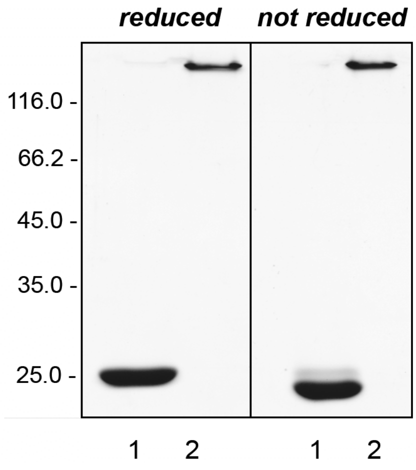

Fig. S2: 12 % SDS-PAGE analysis of PASylated hGH. Lane 1, original recombinant hGH; lane 2, hGH fused with PAS#1(600). Samples on the right are the same but were not reduced with 2-mercaptoethanol. hGH fused with the PAS600 tag exhibits very low electrophoretic mobility in SDS-PAGE (M_W = 73 kDa) as explained in the text.

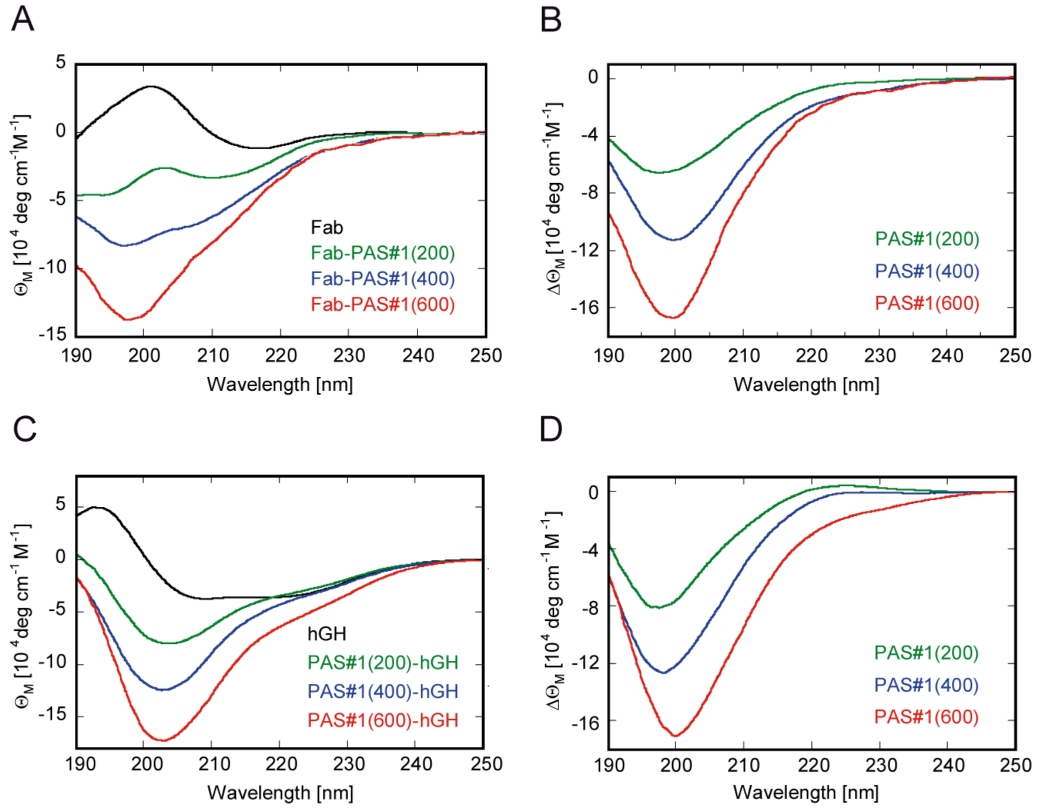

Fig. S3: Secondary structure analysis of PASylated Fab and hGH by CD spectroscopy. Spectra were recorded at room temperature in 50 mM K_2SO_4, 20 mM KP_i pH 7.5 using a 0.01 cm quartz cuvette and normalized to the molar ellipticity, Θ, for each protein. (A) CD spectra of Fab and its PASylated versions with 200, 400, and 600 residues. For all PAS fusion proteins an additional strong minimum around 198 nm appears in these spectra, which is clearly indicative of random coil conformation. (B) Molar difference CD spectra for the PASylated proteins with 200, 400, and 600 residues obtained by subtraction of the spectrum for the unfused Fab from (A). Considered on a molar basis, the negative amplitude at 198 nm increases proportionally to the length of the PAS polypeptide, thus confirming random coil conformation under physiological conditions for the PAS tag. (C) CD spectra of hGH and its PASylated versions with 200, 400, and 600 residues. Again, for all PAS fusion proteins an additional strong minimum around 198 nm appears. (D) Molar difference CD spectra for the PASylated hGH versions with 200, 400, and 600 residues obtained by subtraction of the spectrum for the unfused hGH from (C).

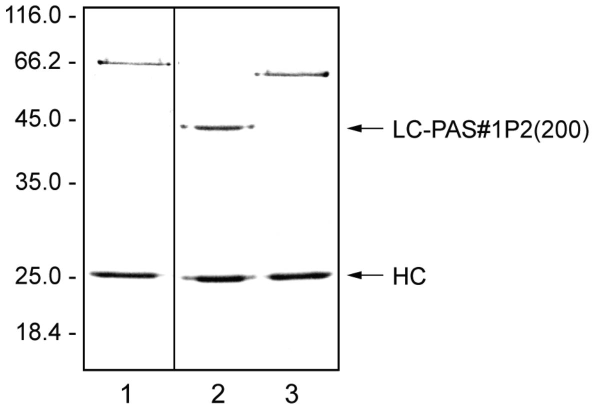

Fig. S4: 12 % SDS-PAGE analysis of the 4D5 Fab fragment fused with different PAS sequences of similar length.

Lanes 1–3, 4D5 Fab fragment fusions with PAS#1(200), PAS#1P2(200), and PAS#5(192), respectively. Samples were reduced with 2-mercaptoethanol. The heavy chain (HC) is labeled by an arrow. The light chain with the PAS#1P2(200) fusion (labeled) exhibits a clearly faster electrophoretic mobility in SDS-PAGE. This behaviour indicates a more compact conformation and/or better binding of SDS compared to the PAS#1 or PAS#5 sequences, illustrating the importance of Pro residues.

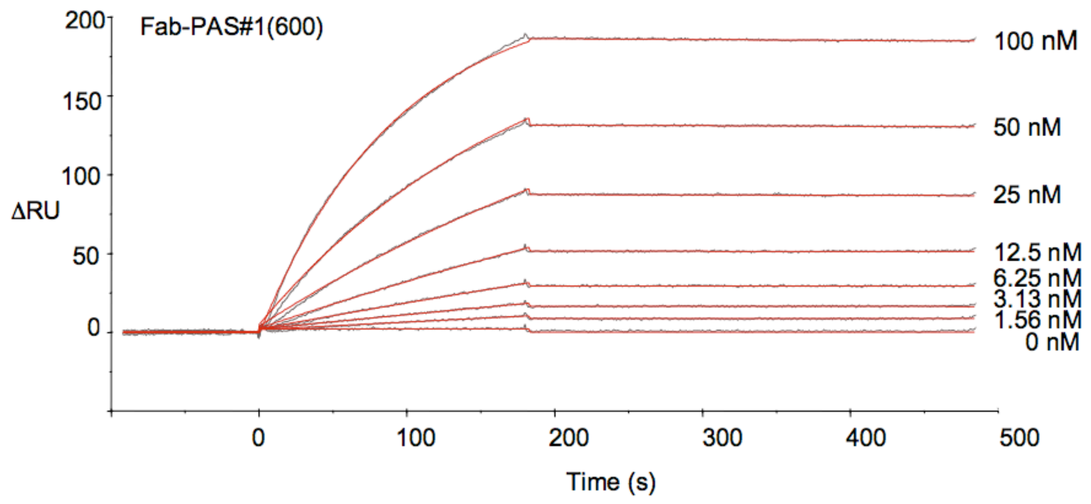

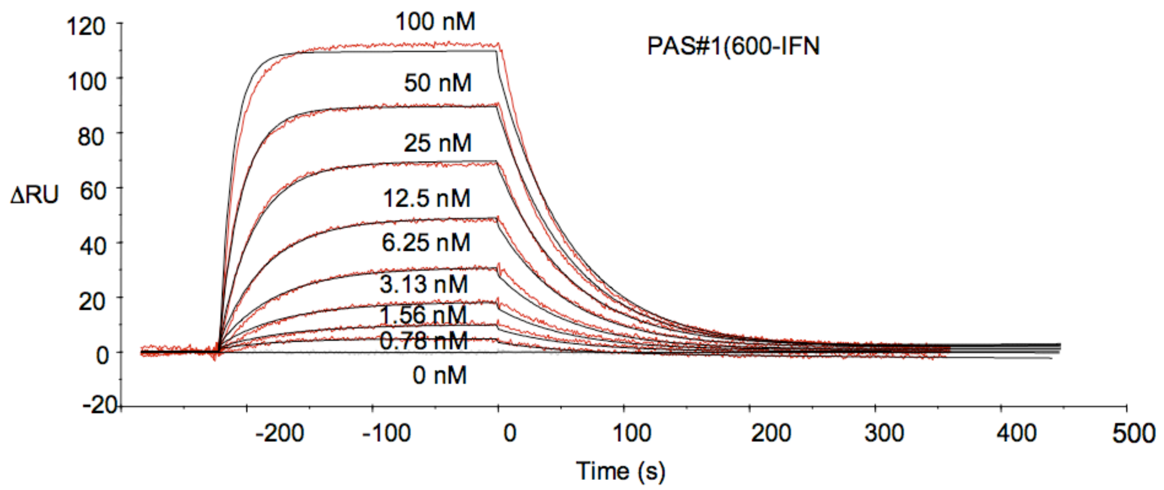

Fig. S5: Real-time Biacore analysis of the PASylated 4D5 Fab fragment and IFN.

Top: The Her2/ErbB2 extracellular domain was coupled via amine chemistry to a CMD200L sensorchip (ΔRU = 540), and the purified Fab-PAS#1(600) was applied at varying concentrations as indicated. Bottom: The recombinant human IFN-α/β R2-Fc chimera was immobilized via an anti-Fc capture antibody to a CMDP sensorchip (ΔRU = 250), and the PAS#1(600)-IFN was applied. The measured signals are shown in red and the curve fits are depicted as a black lines. The kinetic and thermodynamic parameters determined from these curve sets are listed in Table 1. All SPR traces show the typical association and dissociation phases according to the ideal Langmuir model for 1:1 complex formation. In both cases the PASylated protein behaved very similar to the unfused protein and there was only a marginal increase in the dissociation constant (K_D) with growing length of the PAS tag. This was mostly due to a slower rate constant of association (k_{on}), in line with a slightly hampered diffusion through the carboxymethyldextran hydrogel matrix of the sensorchip.

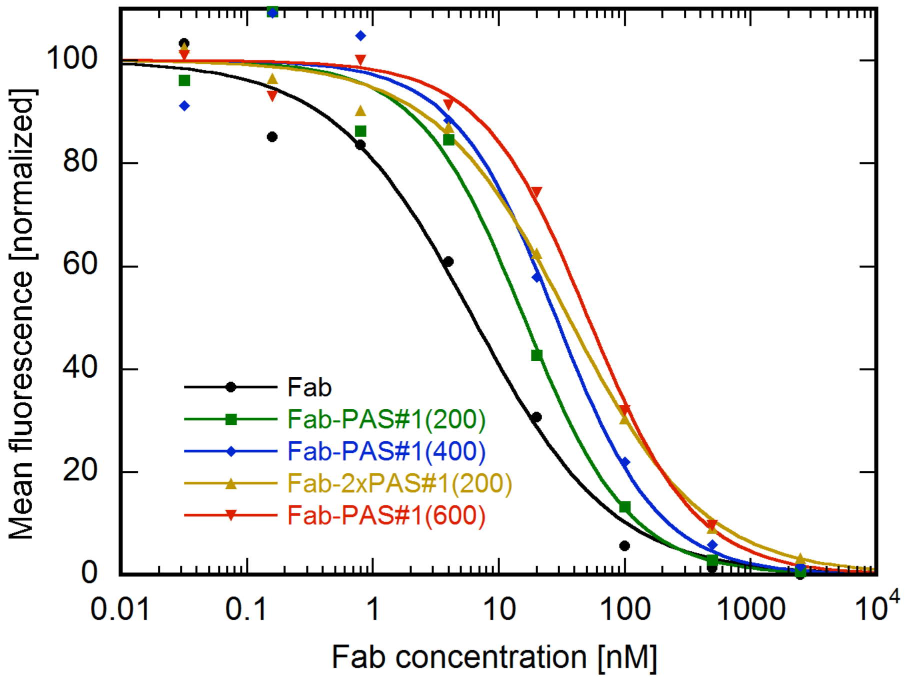

Fig. S6: Competitive cellular receptor binding study of PASylated 4D5 Fab fragments via FACS titration.

Cytofluorimetric analysis of the Her2 overexpressing human breast cancer cell line SK-BR-3 was performed in a FACSAria system using the fluorescence-labelled recombinant 4D5 Fab fragment as tracer at a concentration of 20 nM. The PASylated Fab fragments were applied at varying concentrations from 20 pM to 2 μM for competition. The corrected mean fluorescence data were fitted by a sigmoidal equation and the EC_{50} as well as corresponding K_D values (in brackets) were determined: Fab, 6.3 nM (2.0 nM); Fab-PAS#1(200), 15.9 nM (4.9 nM); Fab-PAS#1(400), 28.5 nM (9.0 nM); Fab-PAS#1(600) 51.3 nM (15.9 nM); Fab-2xPAS#1(200), 36.0 nM (11.2 nM). The measurements reveal a clear competition effect for all PASylated Fab fragments; however, with increasing length (and hydrodynamic volume) of the PAS tag higher EC_{50}/K_D values become apparent, possibly indicating reduced sterical accessibility of Her2 when exposed directly on the plasma membrane and embedded in the glycocalyx.

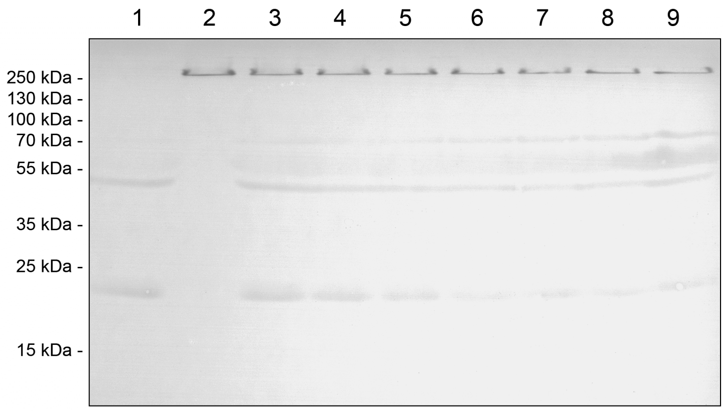

Fig. S7: Analysis of plasma stability for the PASylated hGH.

Purified PAS#1(600)-hGH was incubated in the presence of Balb/C mouse plasma at 37 °C for up to 48 h. Samples taken at different time points were subject to Western blotting, followed by development with an anti-hGH-HRP conjugate. Lane 1, serum only; lane 2, PAS#1(600)-hGH; lanes 3–8: samples taken at 0, 1, 3, 6, 18, 24, and 48 h, respectively. Full length PAS#1(600)-hGH protein is detectable for up to 48 h, demonstrating high stability against serum proteases.

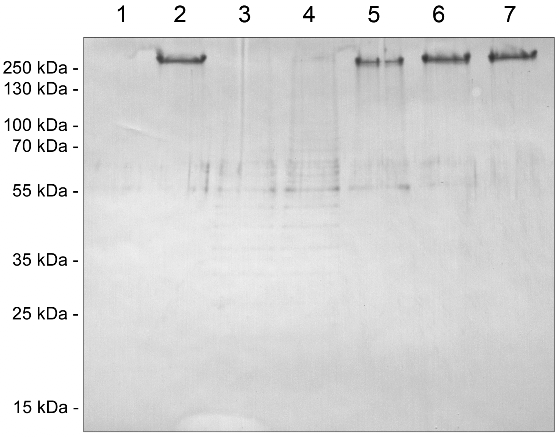

Fig. S8: Assessment of PASylated hGH biodegradability.

Purified PAS#1(600)-hGH was incubated in the presence of a mouse kidney homogenate with varying dilution at 37 °C for 1 h and subjected to Western blot analysis with an anti-hGH-HRP conjugate. Lane 1, 1:5 dilution of homogenate (as control); lane 2, purified PAS#1(600)-hGH; lanes 3–7, PAS#1(600)-hGH incubated with the kidney homogenate at 1:5, 1:10, 1:100, 1:500, and 1:1000 dilution, respectively. The band for PAS#1(600)-hGH disappears with decreasing dilution of the kidney extract, showing that the PASylated protein is rapidly degraded. Thus, in contrast with the poorly degradable chemical polymer PEG, PAS polypeptides should be easily metabolized.

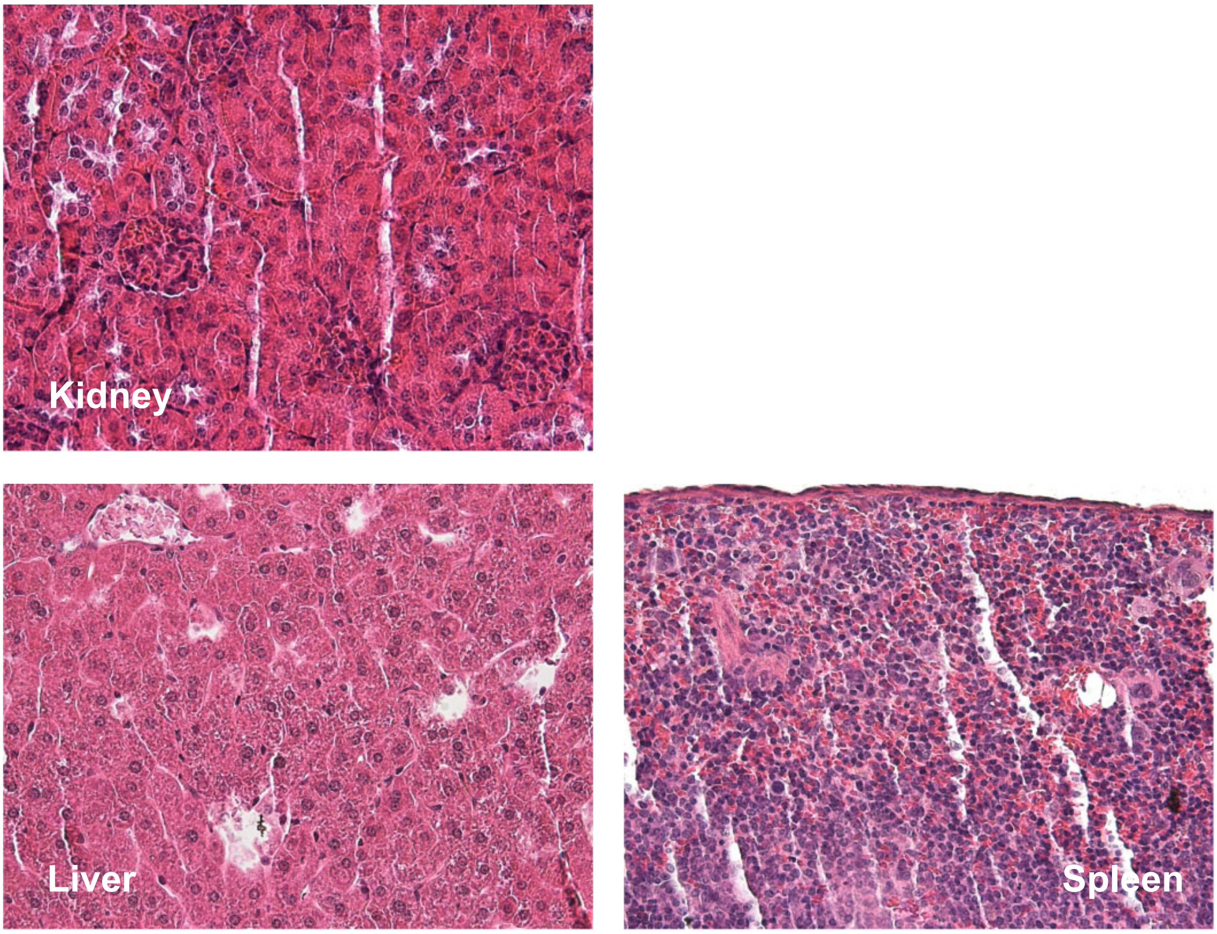

Fig. S9: Histochemical analysis after treatment of mice with PASylated hGH.
After completion of the *in vivo* PD study, involving administration of PASylated hGH over 10 days (cf. Fig. 5C), mice were subjected to histochemical analysis of kidney, liver, and spleen. As an example, typical tissue sections of one mouse are shown. There was no abnormality detected, neither in the mice treated with PAS#1(600)-hGH nor in those receiving the unfused recombinant hGH.

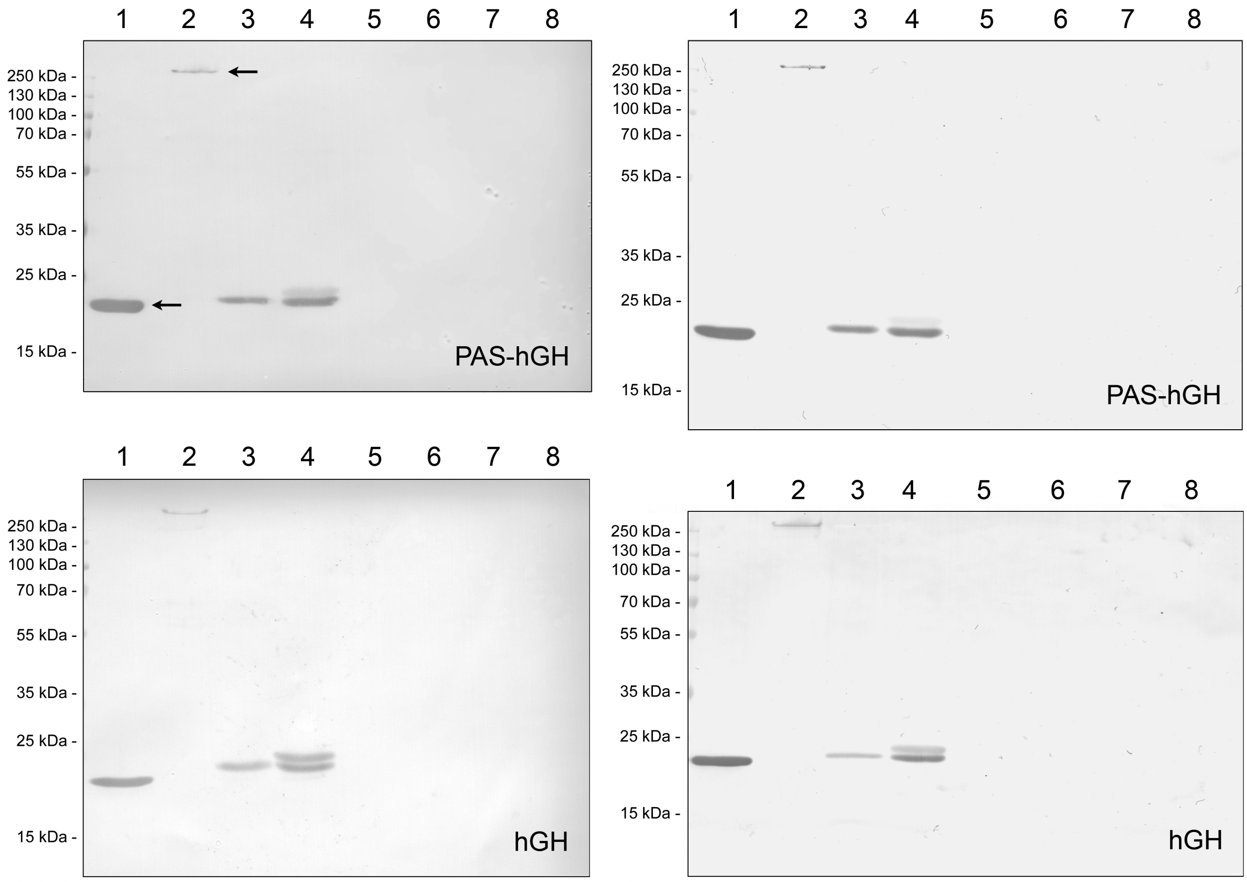

Fig. S10: Immunogenicity analysis of PASylated hGH.

After completion of the *in vivo* PD study, involving administration of PASylated hGH over 10 days (cf. Fig. 5C), mouse sera were probed for immune reactivity. To this end, a set of different test proteins was subjected to Western blotting, followed by incubation with a 1:2500 dilution of sera from individual mice treated either with PAS#1(600)-hGH (upper row) or with the unfused recombinant hGH (lower row): lane 1, hGH; lane 2, PAS#1(600)-hGH; lane 3, 4D5 Fab fragment; lane 4, Fab-PAS#1(600); lane 5, IFN; lane 6, PAS#1(200)-IFN; lane 7, PAS#1(400)-IFN; lane 8: PAS#1(200)-Leptin. Bound antibodies were detected with a goat anti-mouse polyvalent Ig/AP conjugate. While antibodies against hGH were formed in mice treated either with the PASylated protein or with hGH alone (as known in the field), no cross-reactivity against the PAS sequence – as part of other fusion proteins – was detectable. Note that the secondary antibody also seems to recognize the (unfused) heavy chain of the Fab fragment (see the signals in the lower parts of lanes 3 and 4). The arrows depict the hGH band on lane 1 and the PAS-hGH band on lane 2.

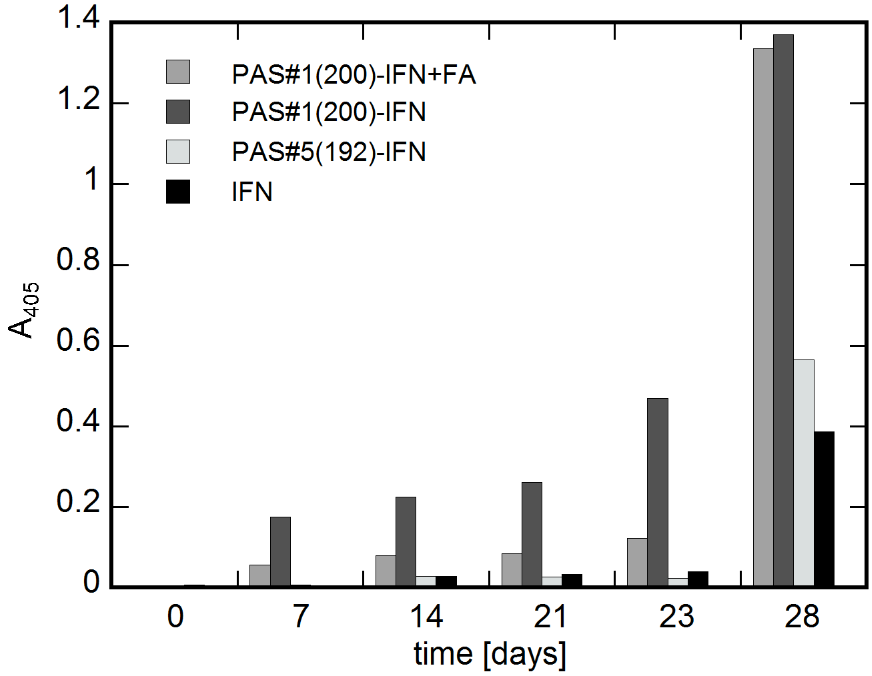

Fig. S11: Immunogenicity analysis of PAS polypeptides fused to human IFNα2b.

Female Balb/C mice were immunized with either PAS#1(200)-IFN, PAS#5(192)-IFN or with the original recombinant IFN with or without Freund's adjuvans (FA). Mice were boosted on day 21 (this time using incomplete FA for the group that had been immunized with the addition of FA). Serum samples of four animals were analysed in an ELISA for antibodies toward the respective antigen to monitor the time course of the immune reaction. For all antigens an immune response was detectable one week after boosting (day 28).

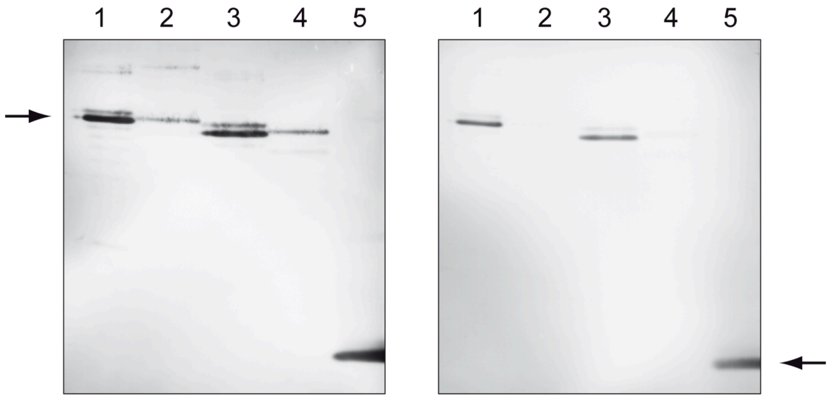

Fig. S12: Immunogenicity analysis of PAS polypeptides by Western-blotting.

To further analyse the immune response detectable for PAS#1(200)-IFN and PAS#5(192)-IFN in Fig. S11 the sera of mouse no. 1 (immunized with PAS#1(200)-IFN+FA) and mouse no. 5 (immunized with PAS#5(192)-IFN) collected on day 28 were probed for immune reactivity. To this end, a set of different test proteins (each 0.6 μg; not reduced) was subjected to SDS-PAGE and Western blotting, followed by incubation with a 1:1000 dilution of sera from mouse no. 1 (left) and mouse no. 5 (right): lane 1, PAS#1(200)-IFN; lane 2, PAS#1(200)-IL-1ra; lane 3, PAS#5(192)-IFN; lane 4, PAS#5(192)-IL-1ra; lane 5, IFN. Bound antibodies were detected with a 1:1000 dilution of an anti-mouse polyvalent Ig/AP conjugate. In both cases, the strongest signals (see arrows) were observed for the unfused IFN and its PASylated versions (lanes 1, 3, and 5). In contrast, much weaker signals appeared for PAS#1 or PAS#5 if fused to the unrelated protein IL-1ra (lanes 2 and 4), indicating low cross-reactivity with either PAS sequence alone. In fact, these residual signals can be explained by antibodies directed against the *Strep*-tag II, which were specifically detected in Fig. S13.

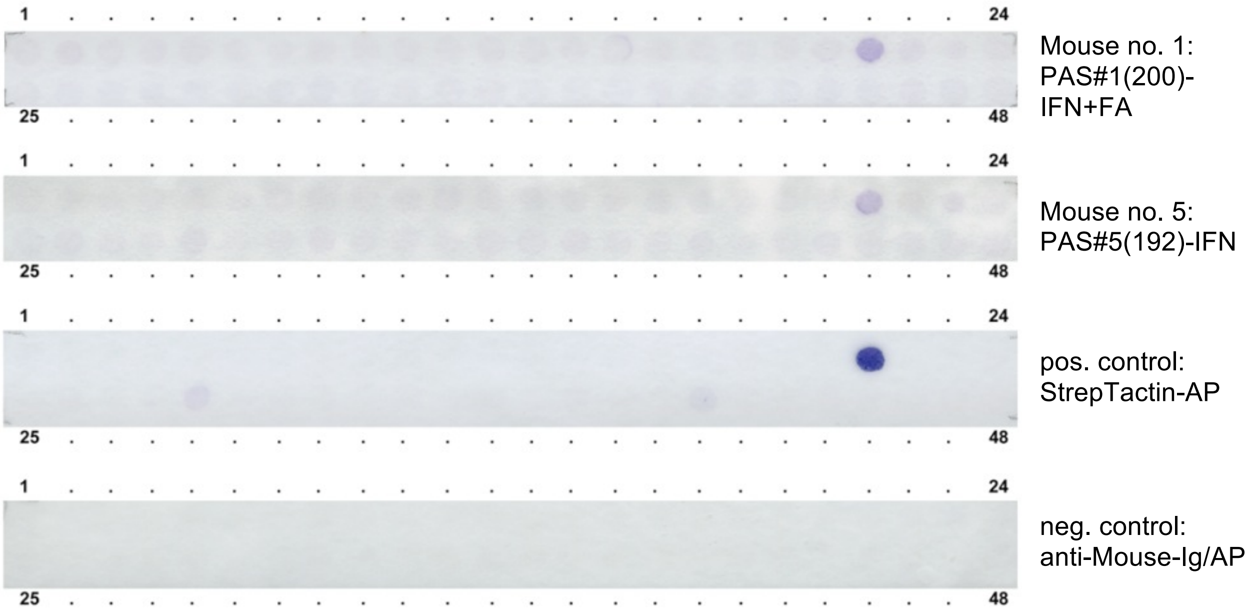

1	ASPAAPAPASPA	13	APAPSAPAASPA	25	AASPAAPSAPPA	37	AASPAAPSAPPA
2	SPAAPAPASPAA	14	PAPSAPAASPAA	26	ASPAAPSAPPAA	38	ASPAAPSAPPAA
3	PAAPAPASPAAP	15	APSAPAASPAAP	27	SPAAPSAPPAAA	39	SPAAPSAPPAAA
4	AAPAPASPAAPA	16	PSAPAASPAAPA	28	PAAPSAPPAAAS	40	PAAPSAPPAAAS
5	APAPASPAAPAP	17	SAPAASPAAPAP	29	AAPSAPPAAASP	41	AAPSAPPAAASP
6	PAPASPAAPAPS	18	APAASPAAPAPA	30	APSAPPAAASPA	42	APSAPPAAASPA
7	APASPAAPAPSA	19	PAASPAAPAPAS	31	PSAPPAAASPAA	43	PSAPPAAASPAA
8	PASPAAPAPSAP	20	AASPAAPAPASP	32	SAPPAAASPAAP	44	SAPPAAASPAAP
9	ASPAAPAPSAPA	21	A**WSHPQFEK**GAS	33	APPAAASPAAPS	45	APPAAASPAAPS
10	SPAAPAPSAPAA	22	HPQFEKGASSSA	34	PPAAASPAAPSA	46	PPAAASPAAPSA
11	PAAPAPSAPAAS	23	FEKGASSSASPA	35	PAAASPAAPSAP	47	PAAASPAAPSAP
12	AAPAPSAPAASP	24	GASSSASPAAPA	36	AAASPAAPSAPP	48	AAASPAAPSAPP

Fig. S13: Epitope analysis of mouse immune sera against PASylated human IFN.

The immune response detectable for PAS#1(200)-IFN and PAS#5(192)-IFN in Fig. S11 was further analyzed in a SPOT assay. To this end, the sera of mice from day 28 were tested using a synthetic peptide spot membrane, which carried covalently immobilized overlapping 12mer peptides covering the entire PAS#1(20) (spots 1–20) or PAS#5(24) (spots no. 25–48) sequence repeats as well the *Strep*-tag II (spot 21, bold sequence) and linker sequences (spots 22–24). Strips 1 and 2 were incubated with the sera of the mice immunized with PAS#1(200)-IFN+FA and PAS#5(192)-IFN, respectively, followed by an anti-mouse Ig/AP conjugate. In both cases only a weak signal against the *Strep*-tag II is detectable. Strip 3 was developed with *Strep*-Tactin/AP conjugate to stain the *Strep*-tag II while strip 4, which was only probed with the anti-mouse Ig/AP-conjugate, served as a negative control. Taken together with the finding from Fig. S12, no immune response was directed against epitopes within the (permuted) PAS#1 and PAS#5 polypeptides, even though the immunized mice had developed antibodies against the human IFN (and also the *Strep*-tag II) – as expected.
